# Wheat gibberellin oxidase genes and their functions in regulating tillering

**DOI:** 10.7717/peerj.15924

**Published:** 2023-09-01

**Authors:** Ting Wang, Junchang Li, Yumei Jiang, Jing Zhang, Yongjing Ni, Peipei Zhang, Ziping Yao, Zhixin Jiao, Huijuan Li, Lei Li, Yufan Niu, Qiaoyun Li, Guihong Yin, Jishan Niu

**Affiliations:** 1 Henan Technology Innovation Centre of Wheat/National Key Laboratory of Wheat and Maize Crop Science, Henan Agricultural University, Zhengzhou, Henan, China; 2 Henan Engineering Research Center of Wheat Spring Freeze Injury Identification, Shangqiu Academy of Agricultural and Forestry Sciences, Shangqiu, Henan, China, Shangqiu, China

**Keywords:** Wheat (*Triticum aestivum* L.), Gibberellin oxidase (GAox), Tillering, Expression profiles, Gibberellin (GA), Gene transformation

## Abstract

Multiple genetic factors control tillering, a key agronomy trait for wheat (*Triticum aestivum* L.) yield. Previously, we reported a *dwarf-monoculm* mutant (*dmc*) derived from wheat cultivar Guomai 301, and found that the contents of gibberellic acid 3 (GA_3_) in the tiller primordia of *dmc* were significantly higher. Transcriptome analysis indicated that some wheat gibberellin oxidase (*TaGAox*) genes *TaGA20ox-A2*, *TaGA20ox-B2*, *TaGA3ox-A2*, *TaGA20ox-A4*, *TaGA2ox-A10* and *TaGA2ox-B10* were differentially expressed in *dmc*. Therefore, this study systematically analyzed the roles of gibberellin oxidase genes during wheat tillering. A total of 63 *TaGAox* genes were identified by whole genome analysis. The TaGAoxs were clustered to four subfamilies, GA20oxs, GA2oxs, GA3oxs and GA7oxs, including seven subgroups based on their protein structures. The promoter regions of *TaGAox* genes contain a large number of *cis*-acting elements closely related to hormone, plant growth and development, light, and abiotic stress responses. Segmental duplication events played a major role in *TaGAoxs* expansion. Compared to *Arabidopsis*, the gene collinearity degrees of the *GAoxs* were significantly higher among wheat, rice and maize. *TaGAox* genes showed tissue-specific expression patterns. The expressions of *TaGAox* genes (*TaGA20ox-B2*, *TaGA7ox-A1*, *TaGA2ox10* and *TaGA3ox-A2*) were significantly affected by exogenous GA_3_ applications, which also significantly promoted tillering of Guomai 301, but didn’t promote *dmc*. *TaGA7ox-A1* overexpression transgenic wheat lines were obtained by *Agrobacterium* mediated transformation. Genomic PCR and first-generation sequencing demonstrated that the gene was integrated into the wheat genome. Association analysis of *TaGA7ox-A1* expression level and tiller number per plant demonstrated that the tillering capacities of some *TaGA7ox-A1* transgenic lines were increased. These data demonstrated that some *TaGAoxs* as well as GA signaling were involved in regulating wheat tillering, but the GA signaling pathway was disturbed in *dmc*. This study provided valuable clues for functional characterization of *GAox* genes in wheat.

## Introduction

Gibberellins (GAs) comprise a large hormone family that modifies many aspects of plant growth and development (Spielmeyer et al., 2004), including seed germination (Yamaguchi & Kamiya, 2001; Urbanova & Leubner-Metzger, 2016), stem elongation (Tian et al., 2022), leaf expansion (Xu et al., 2016), flowering and fruit development (Yu et al., 2004). Currently, more than 136 GAs (Macmillan & Takahashi, 1968; Hedden, 2020) have been identified, and only GA_1_, GA_3_, GA_4_ and GA_7_ are recognized as major bioactive GAs in plants. Therefore, many non-bioactive GAs exist in plants as precursors for the bioactive forms or deactivated metabolites (Yamaguchi, 2008; Hedden & Phillips, 2000).

For more than a century, the biosynthesis and metabolic pathways of GAs have been studied in detail. GAs is biosynthesized from geranylgeranyl diphosphate (GGDP). Three different classes of enzymes are required for the biosynthesis of bioactive GAs from GGDP in plants: terpene synthases, cytochrome P450 monooxygenases, and 2-oxoglutarate-dependent dioxygenases (2ODDs) (Hedden & Thomas, 2012). Among them, three types of GA oxidases (*GAoxs*) in 2ODD gene superfamily are the key enzymes in the synthesis and degradation of GAs (Hu et al., 2021). They are mainly responsible for the mutual transformation of different GAs, and are particularly important in regulating the level of bioactive GAs. GA20-oxidases (GA20oxs) and GA3 Beta-hydroxylases (GA3oxs) are GA biosynthetic enzymes, they convert inactive GAs of GA_12_ and GA_53_ into bioactive GAs of GA_1_, GA_3_, GA_4_ and GA_7_ (Yamaguchi, 2008). In addition, it has been reported that GA7-oxidases (GA7oxs) are also involved in the biosynthesis of GAs. In pumpkin (*Cucurbita maxima* L.) and cucumber (*Cucumis sativus* L.), GA7-oxidases converted GA_12_-aldehyde to GA_12_ efficiently (Frisse, Pimenta & Lange, 2003; Lange et al., 2013). GA2-oxidases (GA2oxs) are recognized as GA deactivation enzymes, they convert GA precursors or bioactive GAs into inactive GAs by 2β-hydroxylation (Thomas, Phillips & Hedden, 1999; Kawai, Ono & Mizutani, 2014). GA1-oxidases (GA1oxs) are GA deactivation enzymes. In cucumber, GA1oxs convert GA_9_ to GA_61_ and GA_4_ to GA_88_, respectively (Lange et al., 2020). In wheat, GA1oxs convert GA_9_ to GA_61_ (Pearce et al., 2015).

So far, *GAox* genes have been identified in many plant species, including Arabidopsis (*Arabidopsis thaliana* L.) (Lange, Krämer & Lange, 2020), maize (*Zea mays* L.) (Ci et al., 2021), rice (*Oryza sativa* L.) (He et al., 2022), soybean (*Glycine max* L.) (Han & Zhu, 2011), jute (*Corchorus capsularis* L.) (Honi et al., 2020), tea plant (*Camellia sinensis* L.) (Pan et al., 2017), grape (*Vitis vinifera* L.) (He et al., 2019), and peach (*Prunus davidiana* L.) (Cheng et al., 2021). The functions of many *GAox* genes have also been elucidated. *AtGA2ox9* contributes to freezing tolerance and *AtGA2ox10* regulates seed production in Arabidopsis (Lange, Krämer & Lange, 2020). Studies of *GA3ox* mutants suggested that bioactive GAs synthesized in the stamens and/or flower receptacles were transported to petals to promote their growth in Arabidopsis (Hu et al., 2008). The *semi-dwarf* gene, *sd1* of rice loses the function of *GA20ox-2*, leading to the *sd1* mutant contained less GA levels than wild-type plants (Ashikari et al., 2002). *OsHox4* gene was involved in GA metabolism, and controlled the expression of *OsGA2ox* and *OsGA3ox* family genes. Overexpression of *OsHox4* gene caused bushy tillers (Zhou, Malabanan & Abrigo, 2015).

Tillering is an important trait of cereal crops that determines spike number, plant type structure, thereby affected the final crop yield (Wang et al., 2019). Tillering is a very complex trait, except the genetic factors and environmental factors, it can also be affected by plant hormones. Some hormones such as IAA (Cai et al., 2018), ABA (Liu & Hou, 2018; Lin et al., 2020), SLs (Nakamura et al., 2013) and CTK (Yang et al., 2020) are directly involved in regulating the tiller bud growth. GAs play promotable and/or inhibitory roles during shoot branching and tillering in different plant species. Several studies showed that GA treatment promoted axillary bud development and shoot branching in woody plants, such as *Jatropha curcas*, papaya (*Chaenomeles sinensis*), hybrid aspen and sweet cherry (*Prunus avium*) (Elfving, Visser & Henry, 2011; Ni et al., 2015a; Rinne et al., 2016). Some studies showed that GA affected tiller bud growth in annual herbaceous plants. In rice, the application of high concentration GAs could promote the degradation of rice OsSHR1 or MOC1, leading to fewer tillers (Lin et al., 2015, 2020). The inhibitory effects of GAs on tiller production were dose-dependent in tall fescue (*Festuca arundinacea* L.) (Zhuang et al., 2019). In bahiagrass (*Paspalum notatum* Flugge), overexpression of *AtGA2ox1* resulted in a significant reduction of the endogenous bioactive GA_1_ contents, and increased the number of vegetative tillers (Agharkar et al., 2007). In tomato (*Solanum lycopersicum* L.), C_19_ GA2oxs silencing led to higher contents of active GA_4_ in axillary buds and few branches (Martínez-Bello, Moritz & López-Díaz, 2015). Overexpressing *OsGA2oxs* increased rice tiller number (Lo et al., 2008). In wheat, *Rht24* encodes TaGA2ox-A9, which confers higher expression of *TaGA2ox-A9* in stems, leading to a reduction of bioactive GAs in stems (Tian et al., 2022).

Previously, we have reported a *dwarf-monoculm* wheat mutant (*dmc*) derived from Guomai 301, and found the content of GA_3_ in the tiller primordia of *dmc* was significantly higher than that in Guomai 301. Transcriptome analysis revealed that GA biosynthetic genes (*TaGA20ox-A2*, *TaGA20ox-B2* and *TaGA3ox-B2*) were lowly expressed, one GAs biosynthetic genes (*TaGA20ox-A4*) and two GAs catabolic genes (*TaGA2ox-A10* and *TaGA2ox-B10*) were highly expressed in the tiller primordia of *dmc* (He et al., 2018; An et al., 2019). It indicates the existence of a negative feedback mechanism that regulates *TaGAox* genes expression. This study was to systematically analyze the roles of gibberellin oxidase genes played during wheat tillering. We identified 63 *TaGAox* genes in wheat genome, and comprehensive analyzed their gene and protein structures, and evolution. The expression profiles of *TaGAox* genes in tiller primordia of Guomai 301 and *dmc* after exogenous GA_3_ application were explored, and the effect of exogenous GA_3_ on wheat tillering was also evaluated. These results were reported here.

## Materials and Methods

### Plant materials and growth conditions

Guomai 301 (Wild type, WT) is a representative semi-winter wheat (Its vernalization requirement is between winter wheat and spring wheat; Vernalization at 0~12 °C for 15~35 days) cultivar in Henan, China. Mutant *dmc* was obtained from ethyl methyl sulfonate (EMS) treated Guomai 301 (Ni et al., 2015b). For GA_3_ treatment, seeds with full grains and the same size were selected, disinfected with 70% ethanol on the surface for 5 min, rinsed with distilled water, and placed in a petri dish for germination at 27 °C. After 48 h, the germinated seedlings were transplanted in experimental field, Henan Agricultural University, Zhengzhou, Henan Province, China (34°51′ N, 113°35′ E, 95 m *a.s.l*.). Each treatment had eight seedlings of Guomai 301 and eight seedlings of *dmc*, they were sown in the same plot, and each treatment had three replicates. For the hydroponics test, the germinated WT and *dmc* seeds were transplanted in tanks with 1.5 L of 1/2 Hoagland nutrient solution, and cultivated in an incubator. The seed germination conditions for genetic transformation were the same as above.

### Identification of TaGAoxs

Wheat genome data and protein data were downloaded from the Ensembl Plants database (IWGSC refseqv1.1, http://plants.ensembl.org/index.html), and were used to identify all members of wheat GAox family. The Hidden Markov Model (HMM) profile of 2OG-FeII_Oxy (PF03171) and DIOX_N (PF14226) were downloaded from the Pfam database (http://pfam.xfam.org). HMMER software (http://www.ebi.ac.uk/Tools/hmmer) was used to search the *TaGAox* genes from wheat genome database. Meanwhile, the protein sequences of Arabidopsis GAox family members, maize GAox family members and rice GAox family members were downloaded from The Arabidopsis Information Resource (TAIR, http://www.Arabidopsis.org), Maize Genetics and Genomics Database (Maize GDB, http://www.maizegdb.org/gene_center/gene) and Rice Genome Annotation Project Database (RGAP, http://plantbiology.msu.edu), and these sequences were used as input sequences to BLASP in the wheat protein database. The output putative GAox protein sequences were confirmed by SMART (http://smart.embl-heidelberg.de) and Pfam searching for the presence of the 2OG-FeII_Oxy and DIOX_N domains. All output protein sequences with e-value ≤ 1e−10 were collected, removing the redundant sequences. The longest transcript sequence corresponding to each candidate gene was selected as the final sequence. Finally, obtained TaGAoxs were named mainly referred in Pearce et al. (2015).

The Expasy Prot Param tool (http://web.expasy.org/protparam) was used to predict the physical and chemical properties of wheat GAox proteins, including amino acid length, molecular weight, and theoretical isoelectric point and so on.

### Construction of the phylogenetic tree

The multiple sequence alignment of wheat, Arabidopsis, maize and rice GAox protein amino acid sequences were carried out using MEGA software (http://www.megasoftware.net). Based on the sequence alignment results, the phylogenetic tree was constructed using Neighbor-joining method in MEGA software, setting the bootstrap parameter to 1,000 and using the default values for other parameters.

### Analyses of conserved motif distributions and gene structures

The online software MEME (http://meme-suite.org) was used to analyze conserved motifs for each TaGAox protein sequences and the maximum number of motif finding was 10. The wheat Generic Feature Format 3 (GFF3) file was downloaded from the wheat genome database and used to elucidate the structure information of the *TaGAox* genes. Illustration depicting of protein motifs, conserved domains and gene structures of *TaGAox* genes was constructed using the TBtools software (Chen et al., 2020).

### Analysis of the *cis*-acting elements

The 2,000 bp upstream sequences before transcription start positions of *TaGAox* genes were extracted from the wheat genome sequence, and the *cis*-acting elements were predicted and analyzed using the PlantCARE (http://bioinformatics.psb.ugent.be/webtools/plantcare/html/).

### Gene duplication and synteny analysis of *TaGAoxs*

Multiple Collinearity Scan toolkit (MCScanX) was adopted to analyze the *TaGAox* gene duplication events, with the default parameters (Wang et al., 2012). Tandem duplication events were defined as chromosomal regions containing two or more genes within 200 kb (Holub, 2001). The synteny relationship of the orthologous *GAox* genes obtained from wheat, Arabidopsis, maize and rice was analyzed, and the syntenic maps were visualized by TBtools (http://github.com/CJ-Chen/TBtools). TBtools was used to calculate the synonymous rate (Ks) and nonsynonymous rate (Ka) substitutions of each duplicated gene pairs and their ratios (Ka/Ks) (Hurst, 2002).

### Tissue specific expression analysis of *TaGAoxs*

The public wheat RNA-Seq datasets were downloaded from the Wheat Expression Browser (http://www.wheat-expression.com). It was used to analyze the expressions of *TaGAox* family genes in different tissues or organs (roots, stems, leaves, spikes and seeds) of Chinese Spring (Table S1). The gene expression level was represented by transcripts per million (TPM). The gene expression values present as log_2_-transformed normalized TPM values and visualized with TBtools.

### GA_3_ treatment and RNA extraction

The seedlings at the three-leaf stage were treated with 2 × 10^−4^ mol/L GA_3_ to analyze the expression patterns of *TaGAox* genes. The tiller primordia of Guomai 301 and *dmc* at 0 h (untreated control), 1 and 2 h after GA_3_ application were sampled respectively. The RNAs of the samples were immediately extracted for gene expression analysis (Zhang et al., 2021).

### qRT-PCR

qRT-PCR was performed as described previously (Zhang et al., 2021). The primers of *TaGAoxs* were designed using primer-blast of NCBI (www.ncbi.nlm.nih.gov/tools/primer-blast). All the primers were listed in Table S2. The *Actin* gene was used as an internal control. Each sample had three biological replicates. The relative expressions of *TaGAoxs* were calculated by 2^−∆∆Ct^ methods (Livak & Schmittgen, 2001). All data were statistically analyzed. The values shown in the form of means ± SD were obtained from three independent experiments (Li et al., 2021).

### Evaluation of the effects of GA_3_ on tillering

The wheat seedlings at the two-leaf stage were sprayed with distilled water and 2 × 10^−4^ mol/L GA_3_ solution respectively on the leaves until all the leaves were wet. Each seedling was sprayed with 5 mL solution, and treated once every 3 days for a total of 10 times. The tiller numbers were observed and counted once every 7 days from the sixth application, when the tiller number was obviously different among different treatments (Li et al., 2021).

### Wheat transformation and expression analysis of *TaGA7ox-A1*

The CDS of *TaGA7ox-A1* was isolated from Chinese Spring. *TaGA7ox-A1* and plant expression vector of pCAMBIA1304 were digested with *Nco* I and *Spe* I. An expression vector pCAMBIA1304-CaMV35S:TaGA7ox-A1 was constructed by inserting *TaGA7ox-A1* under of pCAMBIA1304-CaMV35S promoter, and transformed into *Agrobacterium* GV3101. pCAMBIA1304-CaMV35S:TaGA7ox-A1 was introduced into Guomai 301 by *Agrobacterium* mediated genetic transformation. When the seedlings of Guomai 301 grew to 0.5–1 cm, they were infected by *Agrobacterium* containing the expression vector. Agrobacterium infection solution was diluted to OD_600_ = 0.6 with 100 μmol/L of acetyl syringone. The transformed plants were transferred to a sterilized petri dish and cultured in a dark environment for 48 h. After 30 days of culture at 4 °C for vernalization, they were transplanted into an artificial climate incubator (Dong et al., 2018; Yang et al., 2021; Cao et al., 2021).

Specific primers F2/R2 were designed according to the sequences of the vector and the target gene (*TaGA7ox-A1*), and the positive TaGA7ox-A1-OE transgenic lines were detected by genomic PCR. The leaf DNAs of Guomai 301 and the transgenic plants were extracted at two-leaf stage and tillering stage. The amplified products were sequenced to demonstrate whether *TaGA7ox-A1* gene was successfully transferred into Guomai 301. Under natural tillering conditions, tiller numbers of the transgenic and wild-type lines were observed and recorded every 7 days. The tiller primordia RNAs of Guomai 301 and the transgenic plants were extracted at tillering stage for transgene expression analysis. The primers used in gene isolation, transgenic plant verification and qRT-PCR were listed in Table S2.

## Results

### Identification of TaGAoxs

A total of 64 candidate wheat gibberellin oxidase (TaGAox) proteins were identified by HMMER and BLASTP in wheat Genome. The conserved motif search showed that one of the 64 proteins lacked the DIOX_N domain (TraesCS7D02G046300.1), the other 63 proteins were typical TaGAoxs family members (Table S3). The 63 TaGAox proteins included 13 TaGA20oxs, 3 TaGA7oxs, 6 TaGA3oxs, 40 TaGA2oxs and 1 TaGA1ox. The genes in the four *TaGAox* families were unevenly distributed on wheat chromosomes (Table S3).

The lengths of the amino acid sequences of TaGAoxs ranged from 305 aa (TaGA2ox-A5) to 442 aa (TaGA20ox-A2), with an average of 361.35 aa. The molecular weights of the TaGAoxs ranged from 33.30 to 48.13 KDa, with an average of 39.23 KDa. The TaGA20ox-A2 was the heaviest and TaGA2ox-A5 was the lightest. The predicted values of the isoelectric points of TaGAoxs ranged from 4.92 to 9.19. Among them, 14 TaGAoxs with theoretical pI greater than seven were slightly alkaline, while the remaining TaGAoxs with theoretical pI less than seven were slightly acidic. It was speculated that most of them were acidic proteins. Their aliphatic index ranged from 72.13 to 91.27, with an average of 80.97. For grand average of hydropathicity (GRAVY), TaGA2ox-A16 and TaGA2ox-D16 were two positive negative hydrophobic proteins, the other 61 were hydrophilic proteins (Table S3).

### Phylogenetic tree of wheat GAox proteins

**Figure 1:**
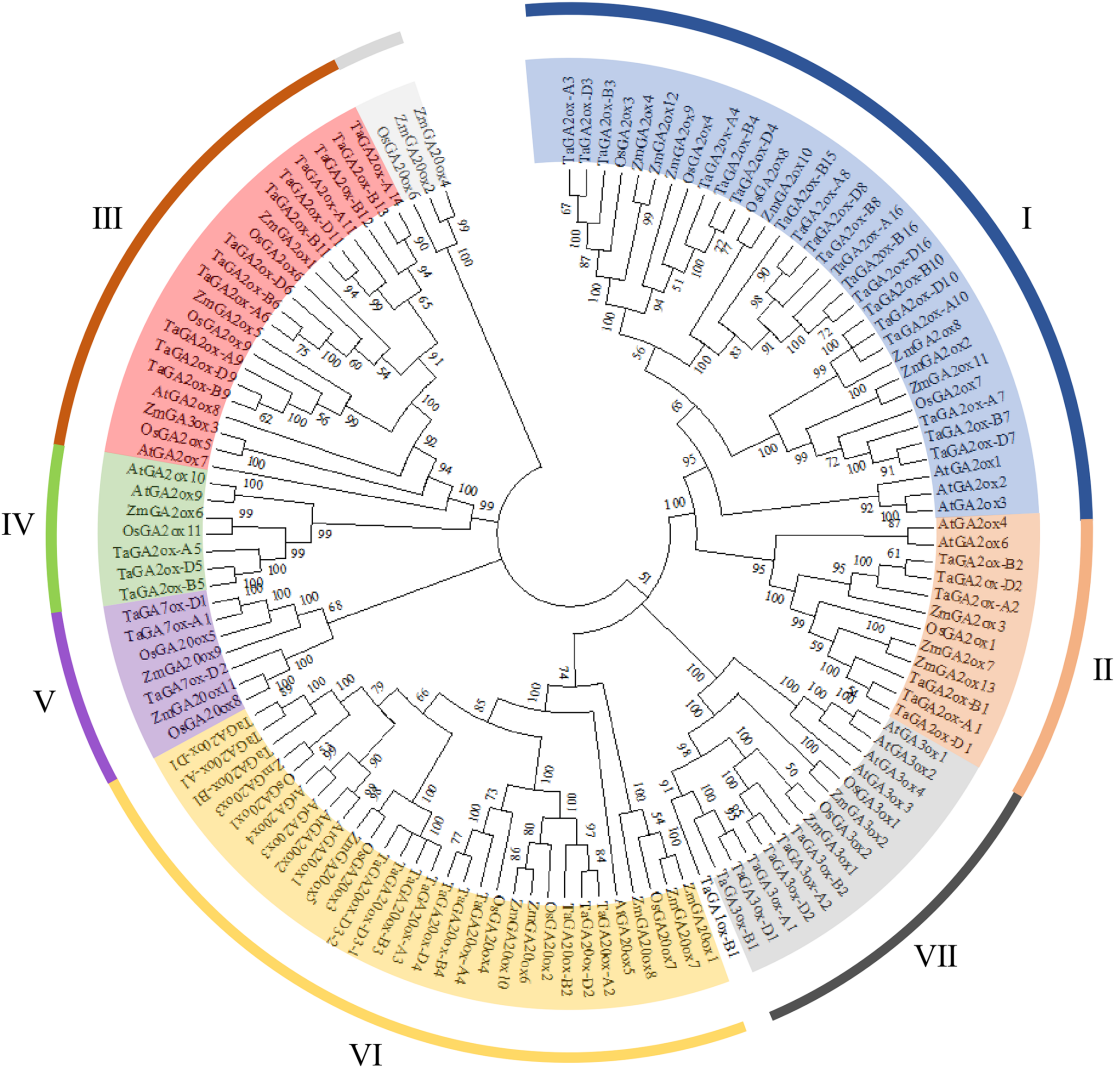
The phylogenetic tree of the GAoxs in *T. aestivum* L. (Ta), *A. thaliana* (At), *Z. mays* (Zm) and *O. sativa* (Os).

To investigate the phylogenetic relationships of TaGAox proteins, the phylogenetic tree was constructed using 127 GA oxidases of wheat, Arabidopsis, maize and rice (Fig. 1). Similar to previous report (Ci et al., 2021), the GAox proteins were clustered to seven distinct subgroups: C_19_-GA2ox (I, II), C_20_-GA2ox (III, IV), GA7ox (V), GA20ox (VI), and GA3ox (VII). They contain 19, 6, 12, 3, 3, 13 and 6 TaGAox proteins respectively. According to previous reports, TaGA1ox-B1 did not fit well into GA3ox subfamily, and TaGA1ox-B1 was shown to encode a GA1-oxidase (Pearce et al., 2015). The evolutionary relationship showed that the distribution of GA20oxs, GA3oxs and GA2oxs in each species was similar. There was no Arabidopsis GA7ox protein in GA7ox (V) subgroup. Most of TaGAox family members were clustered with maize and rice GAox family members. In summary, wheat GAox proteins were more similar to those of maize and rice than those of Arabidopsis.

### Structures and conserved motifs of TaGAox genes

**Figure 2:**
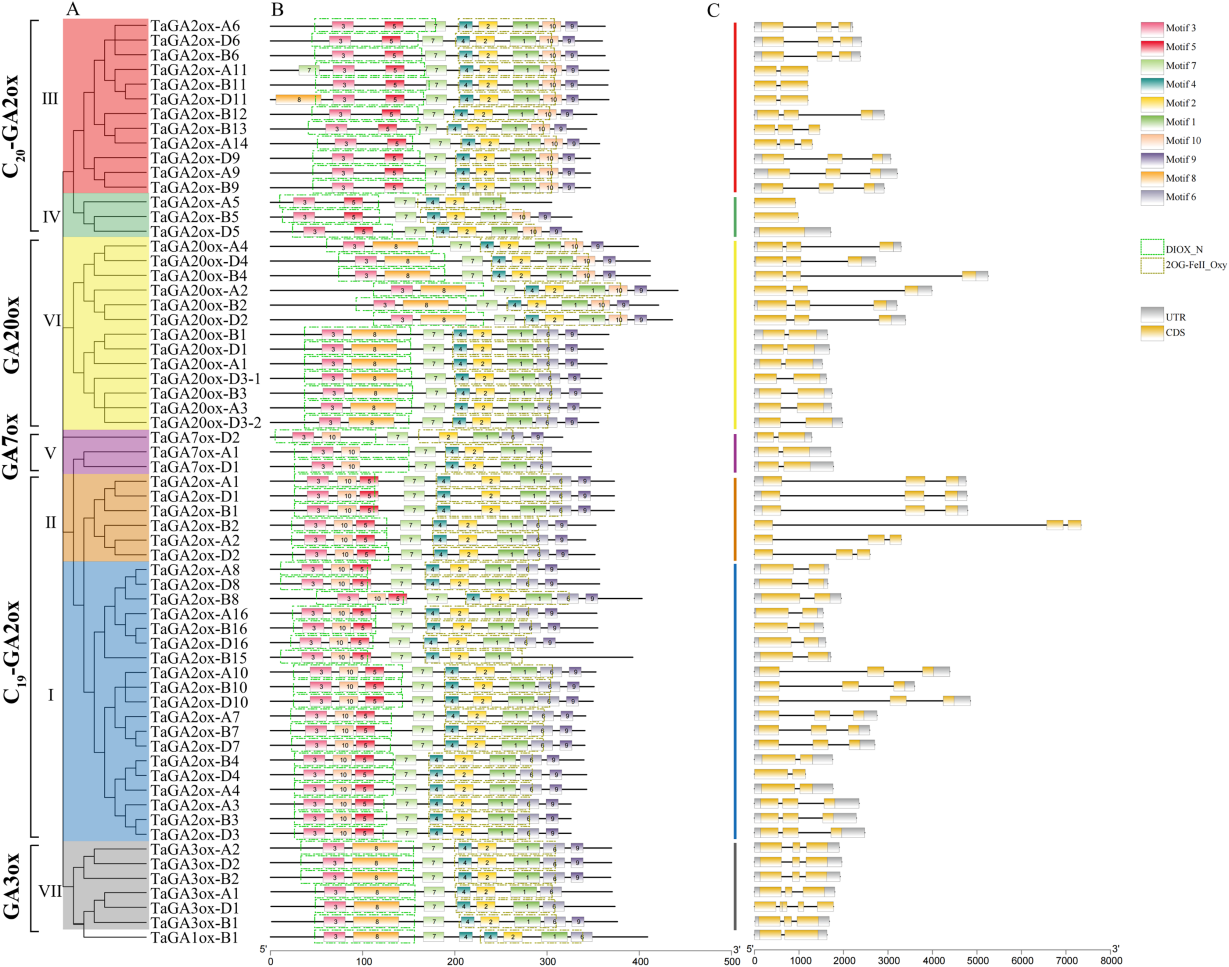
Phylogenetic relationships, conserved protein motif patterns and gene structures of TaGAoxs. (A) The phylogenetic tree of TaGAox proteins. Clusters are indicated with different colors. (B) The motif compositions of TaGAoxs. The 1–10 motifs are displayed in different colored boxes, the scale at the bottom indicates the length of the amino acid sequences. The green dotted boxes represent DIOX_N domains and the brown dotted boxes represent 2OG_FeII oxygenase domains. (C) Exon-intron structures of *TaGAoxs*. Gray boxes indicate 5′- and 3′-untranslated regions; yellow boxes indicate exons; black lines indicate introns.

A phylogenetic tree of TaGAoxs was constructed using the 63 TaGAox protein sequences (Fig. 2A). The clusters of TaGAoxs were basically consistent with those in Fig. 1. To characterize the structures of TaGAox proteins, 10 motifs were identified using the MEME motif search tool (Table S4). The motif compositions and arrangements of the TaGAoxs in the seven subgroups were similar. Among them, motifs 1, 2, 3 and 7 were discovered in all of the TaGAox proteins. Motifs 3, 5 and 8 belonged to the DIOX_N domain, and motifs 1, 2, 4 and 6 belonged to the 2OG_FeII oxygenase domain (Fig. 2B). Motif 5 was unique to TaGA2ox subfamily. Motif 8 was found in GA3ox (VII) and GA20ox (VI) subgroups, and TaGA2ox-D11. Except for TaGA2ox-A5, motif 10 was found in five subgroups of C_19_-GA2ox (I, II), C_20_-GA2ox (III, IV) and GA20ox (VI). A more interesting thing was that motif 10 was located in N-terminal of C_19_-GA2ox (I, II) subgroups and in the C-terminal of C_20_-GA2ox (III, IV) subgroups. The exon-intron structure diagram of *TaGAoxs* showed that their genomic DNA sequence lengths were significantly different (Fig. 2C). The longest was *TaGA2ox-B2*, its length was about 7,500 bp, and the shortest was about 1,500 bp. The exon number of *TaGAoxs* was 1–5, most genes contained two or three exons. *TaGA2ox5* only had one exon, *TaGA3ox-D1* had five exons. As shown in the Fig. 2C, the homoeologous genes had more similar structures, such as *TaGA3ox2* had three exons, which had little difference in structure, length and distribution and might have the same functions.

### *Cis*-acting elements in the promoters of TaGAoxs

**Figure 3:**
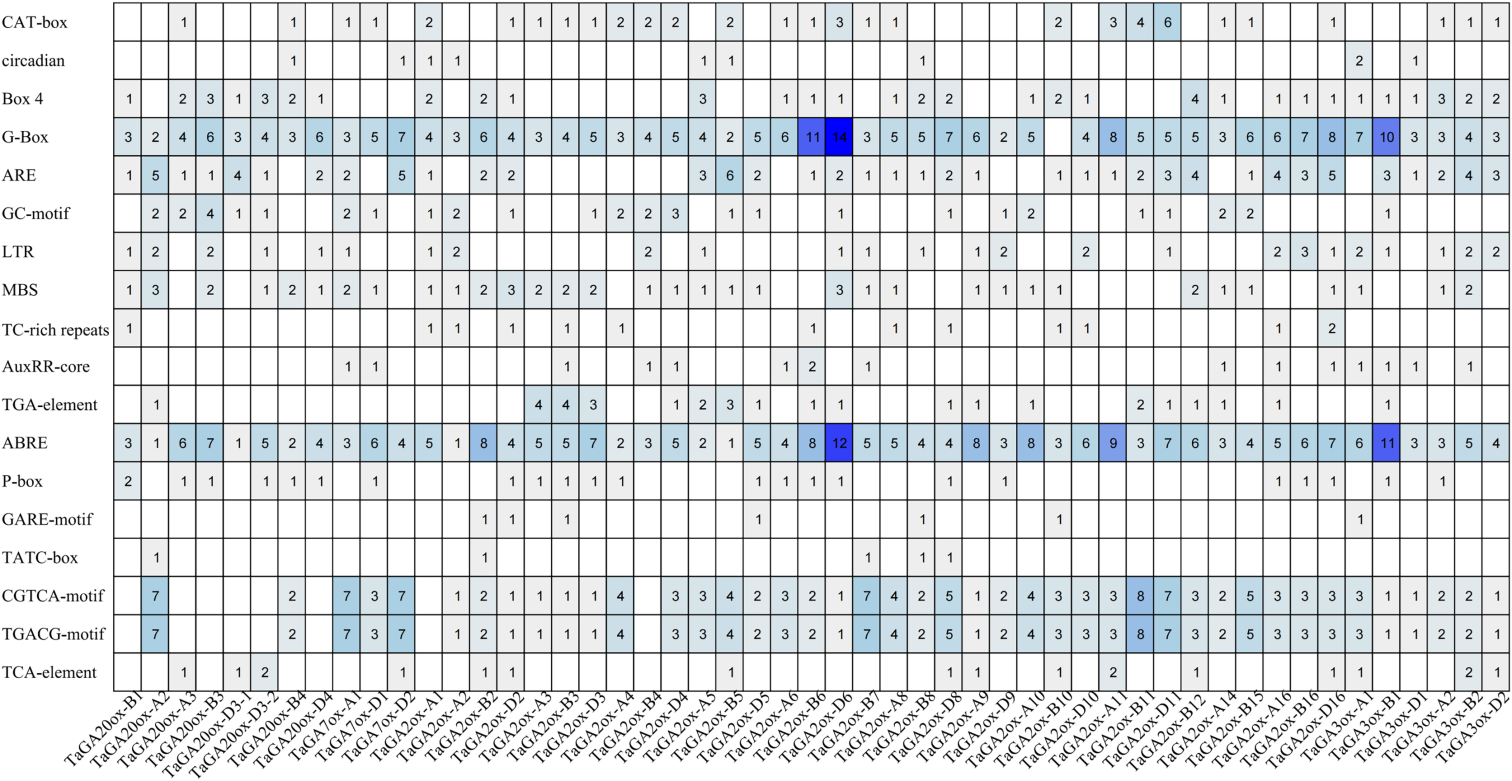
The *cis*-acting elements in the promoters of *TaGAoxs*. The shade of the blue represents the quantity.

To gain a deeper understanding of the potential functions of the TaGAox family genes, we analyzed the *cis*-acting elements in the promoter regions of 51 *TaGAox* genes (the promoter sequences of the other 12 *TaGAox* genes contained a large number of ‘N’, so they hadn’t been analyzed) (Fig. 3). The results showed that there were a large number of *cis*-acting elements related to growth and development, abiotic stress and hormones in the promoter regions of the *TaGAox* genes. Among them, many *cis*-acting elements were involved in auxin response (TGA-element, AuxRR-core); abscisic acid response (ABRE), gibberellic response (P-box, GARE-motif, TATC-box), methyl jasmonate response (CGTCA-motif, TGACG-motif), salicylic acid response (TCA-element, SARE), light response (G-Box, Box 4, Sp1), and those growth-related *cis*-elements (CAT-box, GCN4_motif, O2-site, circadian, RY-element, CCAAT-box) and abiotic stress related elements (ARE, GC-motif, LTR, MBS, TC-rich repeats). The large numbers of elements were those of light-responsive elements, jasmonic acid-responsive elements, and ABA-responsive elements. Although all *TaGAox* genes encoding gibberellin oxidase, some of them did not contain gibberellin response elements, such as, *TaGAox-D2*, *TaGA2ox-A1* and *TaGA3ox-D1*. The existence of various *cis*-acting elements in the gene promoter regions suggested that *TaGAoxs* played important roles in regulating wheat growth and development, stress response and hormone response.

### Synteny relationships of *TaGAoxs*

**Figure 4:**
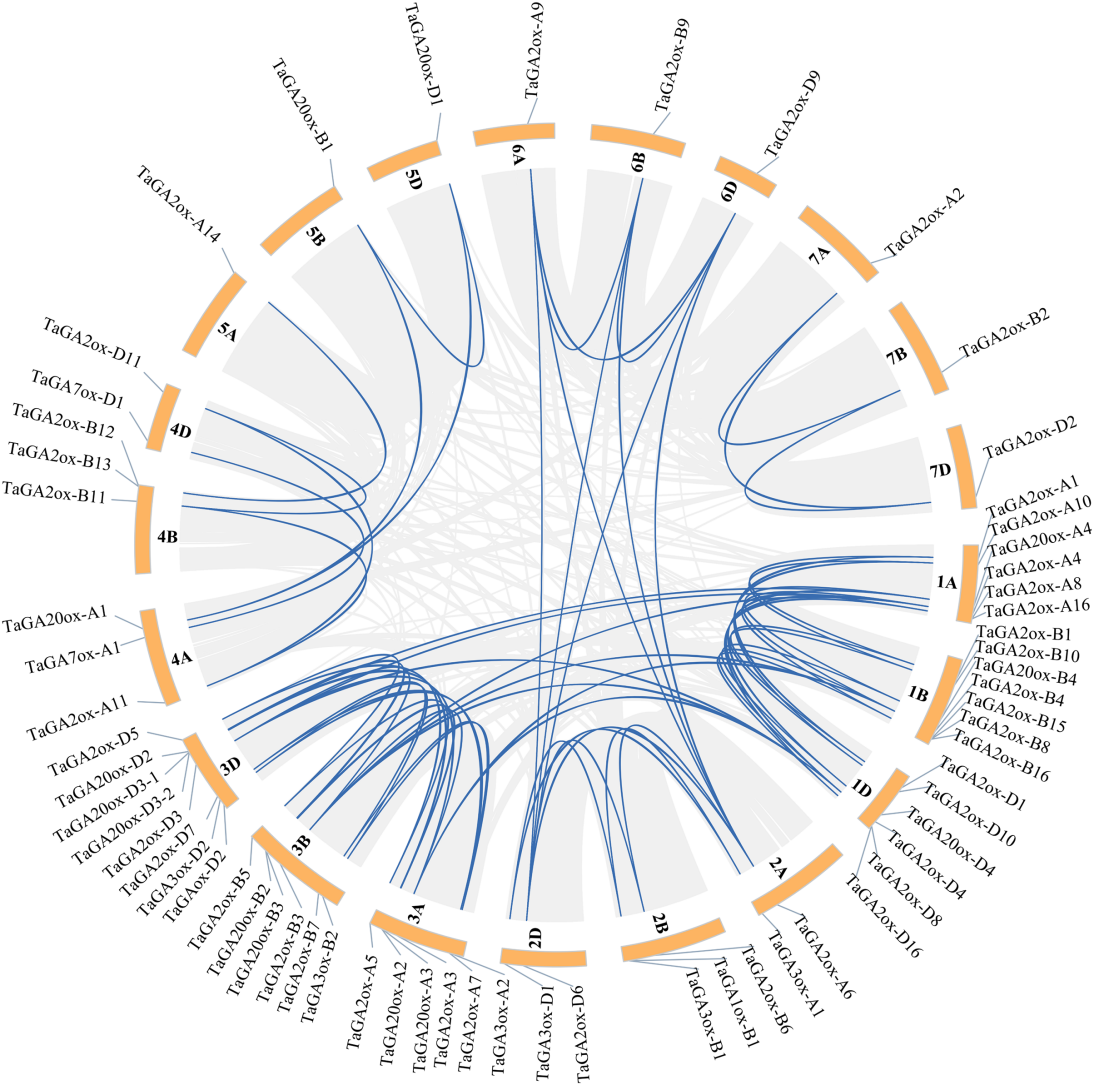
Schematic diagram of the chromosome distribution and inter chromosome relationships of *TaGAoxs*. The gray lines indicate all duplicated gene pairs in wheat, the highlighted blue lines indicate probably duplicated *TaGAox* gene pairs.

During plant evolution, whole-genome duplications, transpositions, tandem gene duplications and segmental duplications play important roles in gene expansion and generation (Hu et al., 2021). In order to discover the gene duplication events of *TaGAoxs*, the 63 *TaGAox*s were investigated. A total of 74 duplicated gene pairs were discovered, including 72 segmentally duplicated gene pairs, they distributed on different chromosomes (Fig. 4, Table S5). According to the methodology of Holub (Holub, 2001), there were two pairs of tandem duplication genes (Table S5). These results indicated that the expansions of *TaGAox* genes were mainly segmental duplications or tandem duplications, and the segmental duplication events played major roles in *TaGAox* genes evolution. To better determine the selective evolutionary pressure on *TaGAox* gene divergence, we calculated the ka/ks ratios of all the syntenic gene pairs. The ka/ks ratios of *TaGA2ox4-1A* and *TaGA2ox4-1B-1* were 1.037, indicating that they had undergone neutral evolution, but the ka/ks ratios of the other 73 pairs of replicated genes were less than one (Table S6), indicating that the *TaGAox* genes mainly underwent purifying selection.

**Figure 5:**
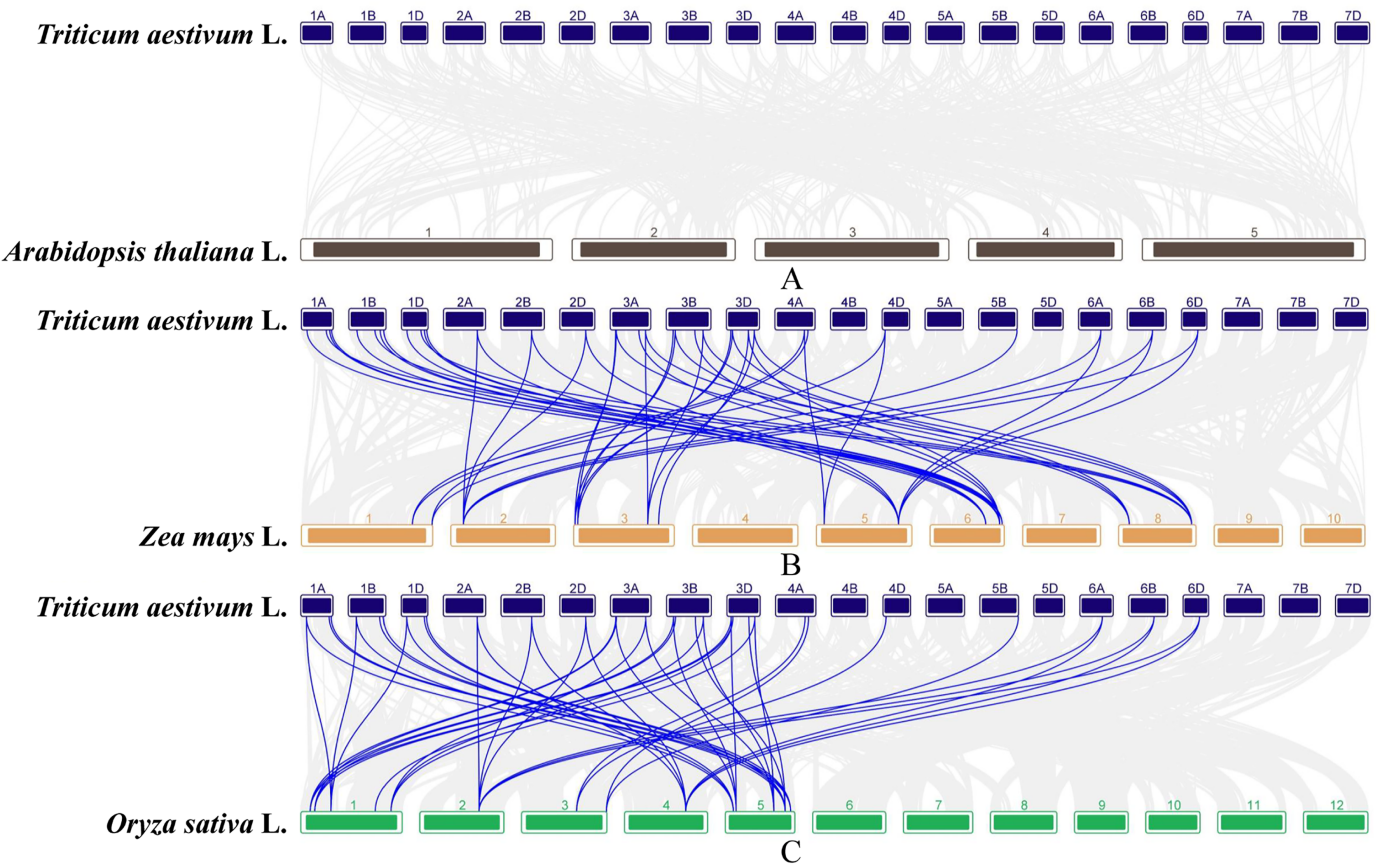
(A–C) Syntenic relationships of *GAox* genes between wheat and three representative species. Gray lines in the background indicate the collinear blocks within wheat and other plant genomes, while the blue lines highlight the syntenic *GAox* gene pairs.

In order to further infer the evolutionary relationships of the *GAox* genes, the comparative syntenic maps associated with wheat genome were constructed with other species, including Arabidopsis, rice, and maize. No syntenic gene of wheat *GAox* genes was found in Arabidopsis (Fig. 5A). A total of 30 *TaGAox* genes had syntenic genes in rice (Fig. 5B) and 34 *TaGAox* genes had syntenic genes in maize (Fig. 5C). The number of the orthologous gene pairs between wheat and rice, wheat and maize were 43 and 47, respectively. The ka/ks ratios of these gene pairs (Table S7) were calculated. All *GAox* gene pairs had ka/ks < 1, suggesting that *TaGAox* genes had undergone a strong purification selection pressure.

### The expression patterns of *TaGAoxs* in different tissues

**Figure 6:**
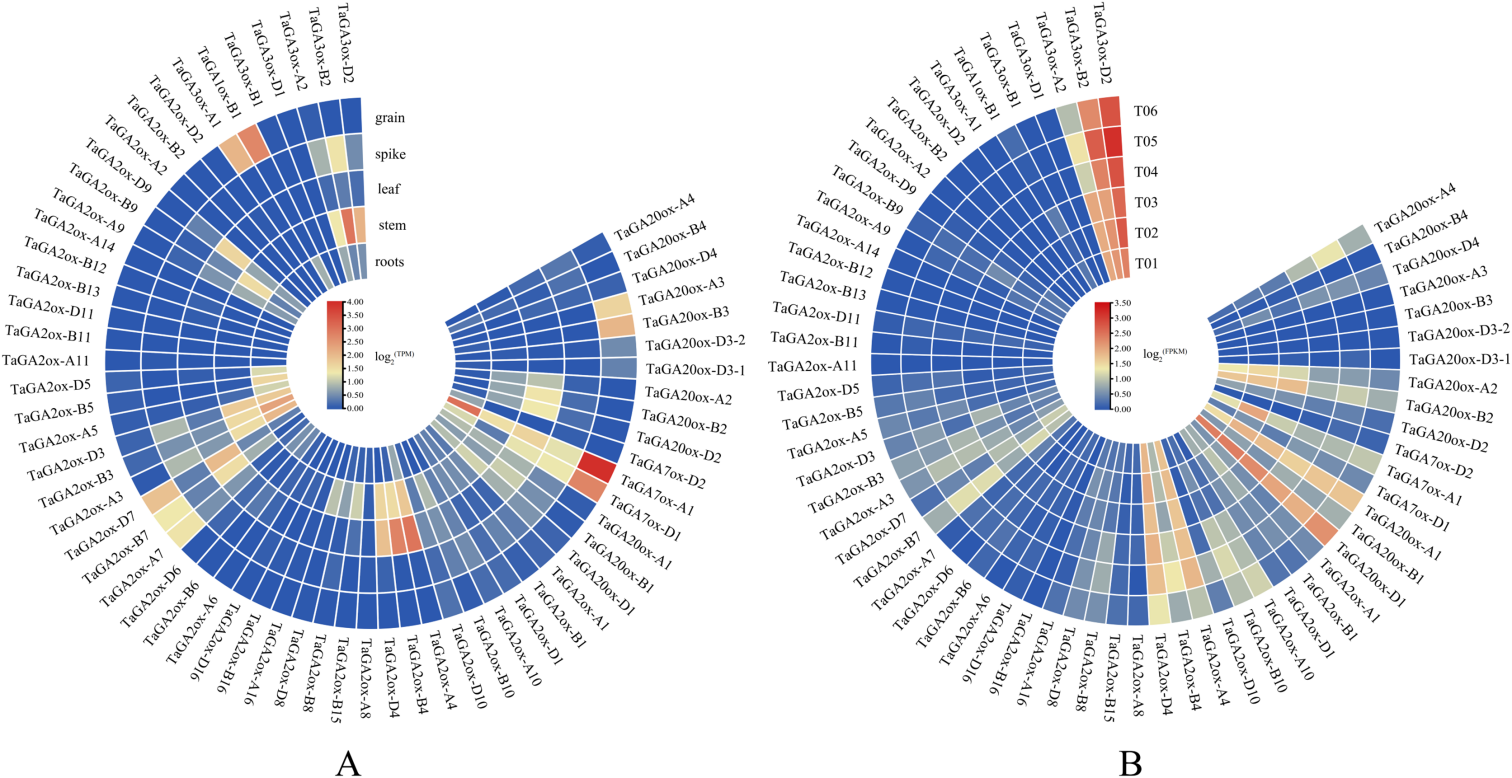
Expression profiles of *TaGAoxs* in various organs or tissues. (A) Heatmap of expression profiles of *TaGAoxs* in various organs or tissues of Chinese Spring. (B) Heatmap of expression profiles of *TaGAoxs* in tiller primordia of Guomai 301 and *dmc* based on transcriptome data. Three biological replicates were set up in Guomai301 (T01, T02 and T03) and *dmc* (T04, T05 and T06). The gene expression values present as log_2_^(FPKM)^. Note: Blue, Low expression level; Red, High expression level.

To gain insight into the putative functions of *TaGAox* genes, the expression profiles of *TaGAoxs* were analyzed using public RNA-Seq data from different organs/tissues of wheat (Fig. 6A, Table S1). Because it expressed at relative high levels in all tested tissues, *TaGAox1* might play an important role during wheat development. *TaGA2ox4*, *TaGA2ox3* and *TaGA3ox2* were highly expressed in stem. *TaGA20ox2* and *TaGA2ox4* were highly expressed in spikes. Most of *TaGAox* genes were lowly expressed or not detected in the five tissues, implying functional redundancy of the *TaGAox* genes. The above results indicated that different *TaGAox* genes may be involved in different growth and development processes of wheat. The expressions of some genes were tissue-specific, which would contribute to different morphogenesis in plant development.

Based on our published transcriptome data (He et al., 2018), the expression profiles of all the *TaGAox* genes in Guomai 301 and *dmc* were further analyzed (Fig. 6B, Table S8). The expression profiles of *TaGAoxs* in tiller primordia showed that most genes expressed very lowly in all detected samples, these genes probably were not necessary for wheat tiller development. *TaGA3ox-B2* and *TaGA3ox-D2* were highly expressed in Guomai 301 and *dmc*. High expression levels suggested they played important roles during tiller development. Compared to WT, *TaGA20ox-A4*, *TaGA2ox-A10* and *TaGA2ox-B10* were highly expressed, and *TaGA20ox-A2*, *TaGA20ox-B2* and *TaGA3ox-A2* were lowly expressed in *dmc*. Differential expression of these genes might be one of the main causes containing tillering of *dmc*.

### Expression patterns of *TaGAoxs* in response to GA_3_ application

**Figure 7:**
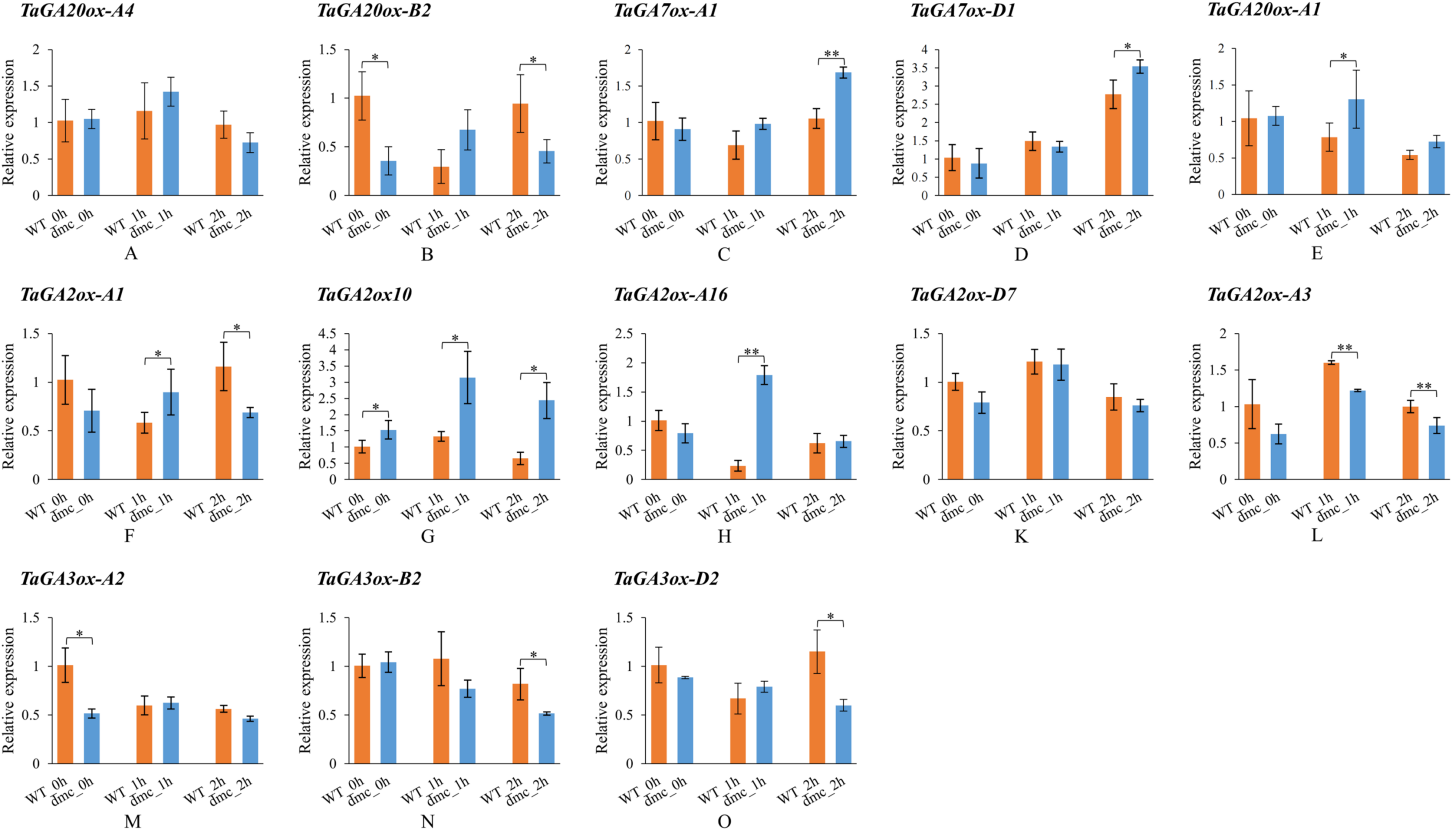
Expression profiles of *TaGAoxs* in response to GA_3_ stimulation. WT_0h, *dmc*_0h: untreated controls; WT_1h, *dmc*_1h: 1 h after GA_3_ treatments; WT_2h, *dmc*_2h: 2 h after GA_3_ treatments. Data were normalized to *Actin* gene and vertical bars indicated standard deviation. Asterisks indicate significant difference or highly significant difference between WT and *dmc*. An asterisk (*) and two asterisks (**) indicate significant difference (*P* < 0.05) and highly significant difference (*P* < 0.01) using Student’s *t*-test, respectively.

In order to explore whether the expressions of *TaGAox* genes in wheat tiller primordia could be activated by GA_**3**_. Some *TaGAox* genes were selected to test according to their expression levels in *dmc* (Fig. 6B). It was found that GA_**3**_ significantly affected the expressions of *TaGAoxs* (Fig. 7). In *dmc*, the expressions of *TaGA2ox* genes were highest at 1 h after GA_3_ treatment. The expressions of *TaGA3ox-B2* and *TaGA3ox-D2* continuously decreased and those of *TaGA7ox-A1* and *TaGA7ox-D*1 continuously increased. The expressions of *TaGA20ox-B2* and *TaGA3ox-A2* had no significant changes. The expressions of *TaGA20ox-A4* and *TaGA20ox-A1* were down-regulated at 2 h after GA_3_ treatment. The expression profiles of *TaGAoxs* in WT were different from those of *dmc*. The expressions of six *TaGAoxs* had no significant changes after GA_3_ treatment. The expressions of *TaGA2ox-A1* and *TaGA20ox-B2* decreased at 1 h after GA_3_ treatment, and recovered after 2 h. In both WT and *dmc*, *TaGA7ox-D1*, *TaGA2ox10*, and T*aGA2ox-A16* were very sensitive to GA_3_ stimulation, the reason was considered as there were *cis*-acting elements of gibberellin response in their promoter regions. Although the gibberellin *cis*-acting elements of the *TaGAox*s may be the same, most expression profiles of *TaGAox*s in WT and *dmc* were significantly different.

In summary, after GA_3_ application, the expressions of GAs catabolic genes and *GA7ox* genes were up-regulated, the expressions of GA biosynthetic genes were down-regulated.

### Effects of GA_3_ on tiller formation of wheat

**Figure 8:**
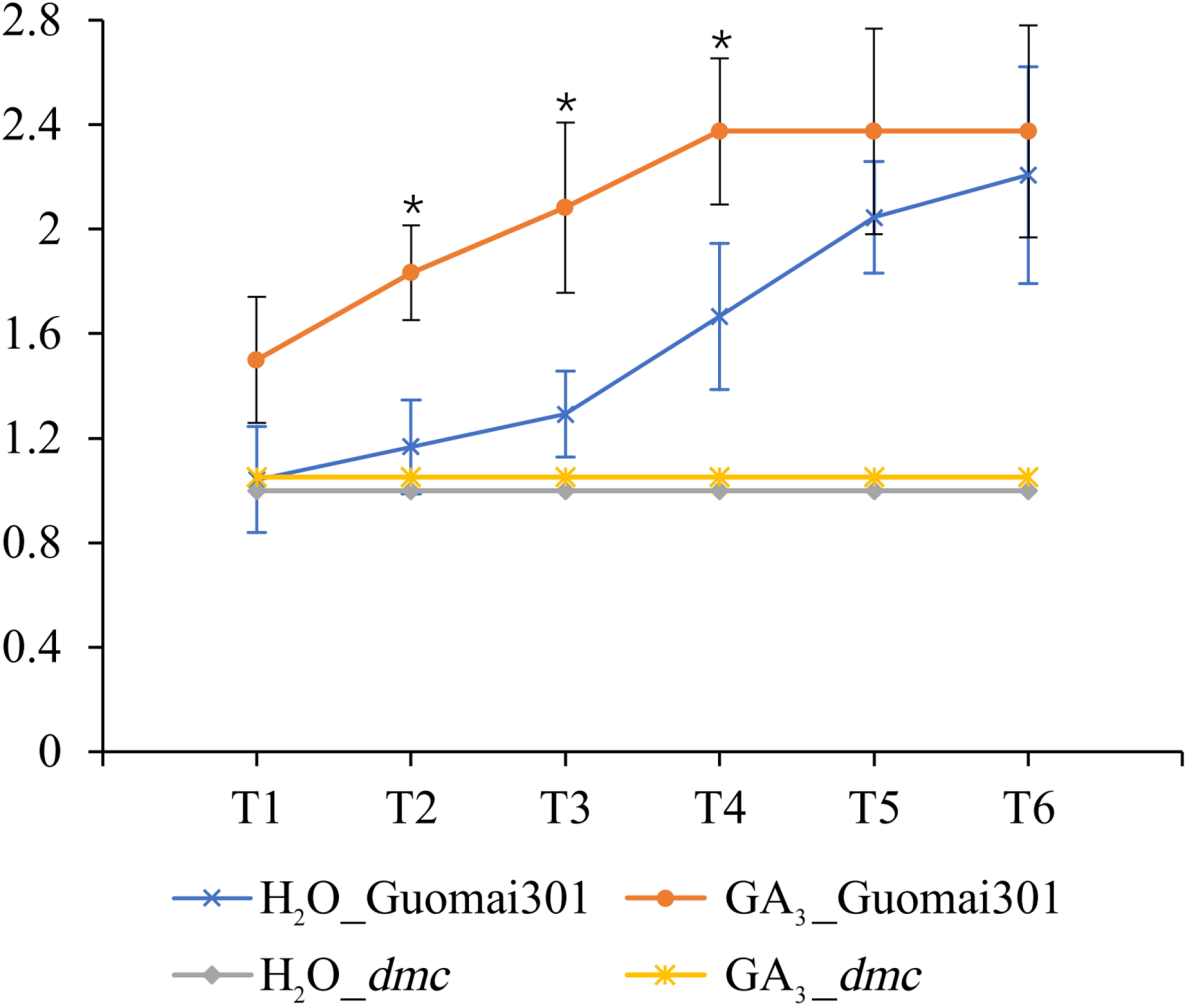
Tiller number changes of Guomai 301 and *dmc* in response to GA_3_ stimulation. T1–T6: the GA_3_ treatment time points, the intervals were 7 days. An asterisk (*) indicate significant difference (*P* < 0.05) using Student’s *t*-test.

After the sixth time of GA_3_ application, the tiller number of Guomai 301 began to appear differences. Compared to the control, continuous GA_3_ treatment significantly increased the tiller number of Guomai 301, but didn’t affect tillering of *dmc* (Fig. 8). The results indicated that the exogenous GA_3_ could significantly promote tiller development of Guomai 301, but the *dmc* lacked response to GA_3_.

### Function of *TaGA7ox-A1* in regulating wheat tillering

**Figure 9:**
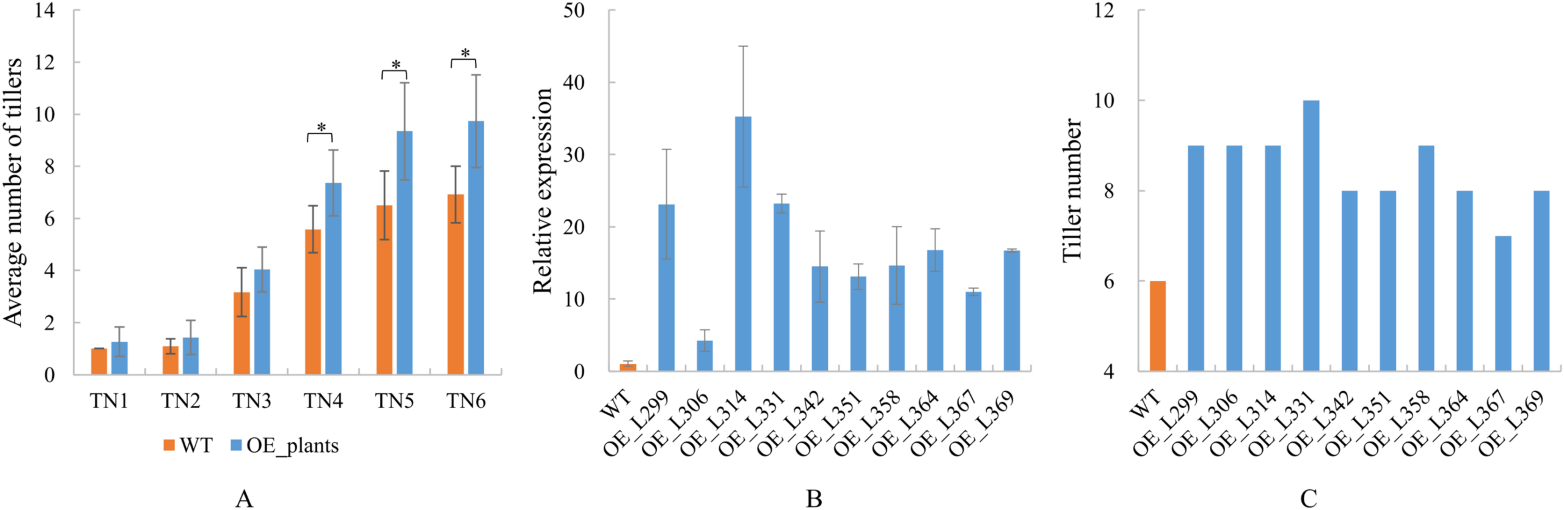
The tiller numbers and qRT-PCR analysis of *TaGA7ox-A1*-OE transgenic lines. (A) Average tiller numbers of the transgenic plants and controls (WT) at different stages. TN1–TN6: time points of the tiller number record, the intervals were 7 days. (B) *TaGA7ox-A1* expression levels of the transgenic plants and WT. (C) Tiller numbers of the transgenic plants and WT (TN4 stage). An asterisk (*) indicate significant difference (*P* < 0.05) using Student’s *t*-test.

Overexpression transgenic plants of *TaGA7ox-A1* were obtained (Table S9). The positive transgenic plants were confirmed by PCR at early tillering stage and the amplified target fragments were sequenced at the late tillering stage (Fig. S1). These results indicated that the *TaGA7ox-A1* was transferred into the positive transgenic plants (Fig. S2). Statistical analysis indicated that the average tiller numbers of *TaGA7ox-A1*-OE transgenic plants were significantly higher than those of WT from the TN4 stage. At the TN6 stage, the tiller number of wild-type plants was 6–7, while the tiller number of transgenic plants was 9–10 (Fig. 9A, Table S9). Compared to WT, the *TaGA7ox-A1* gene relative expression levels and the tiller numbers of the transgenic plants increased significantly (Figs. 9A, 9B, Table S9). The wheat tiller number was significantly correlated with *TaGA7ox-A1* gene expression level (*r* = 0.613, *P* < 0.05) (Table S9). This result indicated that *TaGA7ox-A1* was involved in regulating wheat tillering.

## Discussion

### Characteristics and evolution of wheat *GAox* gene family members

Gibberellins are important hormones in plants, and act during the whole life cycle of plants (Hernández-García, Briones-Moreno & Blázquez, 2021). In model plant Arabidopsis, the signaling pathways related to GAs have been well elucidated, and gibberellin oxidases are involved in the last step of GA biosynthesis pathway (Yamaguchi, 2006). However, the specific functions of the most *GAox* genes are largely remained unknown. Genome-wide predictions of *GAox* genes have become possible when many plants genomic sequences have been reported. In this study, we analyzed the structure, phylogenetic relationships, chromosomal locations, gene duplication events, *cis*-elements, and expression patterns of *GAox* genes in wheat.

A total of 63 TaGAox proteins with typical conserved domains, 2OG-FeII_Oxy and DIOX_N, had been identified in this study. Similar to other plant GAox proteins in Arabidopsis, rice, and soybean (Han & Zhu, 2011), the TaGAox proteins belonged to the 2OG-Fe (II) oxygenase superfamily. In this study, 13 TaGA20oxs, 40 TaGA2oxs, 6 TaGA3oxs, 3 TaGA7oxs and 1 TaGA1ox were identified, respectively. The preliminarily identified *TaGAox* genes included 10 *TaGA20oxs*, 29 *TaGA2oxs*, 6 *TaGA3oxs* and 1 *TaGA1ox*, and they were proved to have the corresponding oxidase activities (Pearce et al., 2015). Kumagai et al. (2022) identified 13 *TaGA20oxs*, this result was consistent with that of this study. Previous research identified the full-length genes of *TaGA20ox2* and *TaGA20ox3* in B and D genomes, but only partial sequences in A genome. In this study, the newly identified *TaGA20ox-A2* and *TaGA20ox-A3* were their homologous genes in A genome. The sequences of *TaGA20ox-D3-2* and *TaGA20ox-D3-1* were highly similar, and they were homologous genes. A study indicates OsGA20ox5 and OsGA20ox8 were clustered into GA7ox subfamily with CsGA7ox1 and CsGA7ox2 (Huang et al., 2015; Kawai, Ono & Mizutani, 2014; Sun et al., 2018). In this study, 3 TaGA7oxs and OsGA20ox5 and OsGA20ox8 are divided into subgroup V. So far, GA7ox activity was reported in pumpkin and cucumber, but has not been found in other species (Frisse, Pimenta & Lange, 2003; Lange et al., 2013). Therefore, whether the three *TaGA7oxs* have GA7ox activity needs to be further verified.

Compared to previous studies (Pearce et al., 2015), 11 *TaGA2ox* genes were newly identified in this study. Among them, *TaGA2ox-A2*, *TaGA2ox-A7*, *TaGA2ox-A8* and *TaGA2ox-D8* were homologous genes of *TaGA2ox2*, *TaGA2ox7* and *TaGA2ox8*. Their homologous genes were used to prove that they have reduced substrate specificity for C_19_-GAs, as it effectively converted GA_12_ to GA_110_ as well as GA_9_ to GA_51_ (Pearce et al., 2015). It is speculated that these four newly identified *TaGA2ox* genes also have similar activities. In addition, TaGA2ox5 and AtGA2ox9-10 were divided into subgroup IV (C_20_-GA2ox) (Lange, Krämer & Lange, 2020). *TaGA2ox15* and *TaGA2ox16* and *TaGA2ox8* were paralogous genes. Whether these newly identified *TaGAox* genes have the activity of GA2-oxidases oxidase needs further study.

Some reports indicated that the enzyme specificities of a few GAoxs were different from that of predicted by amino acid sequences, however, the enzyme activities were yet gibberellin oxidases (Frisse, Pimenta & Lange, 2003; Sun et al., 2018; Lange, Krämer & Lange, 2020; Lange & Lange, 2020). One of the reasons may be that some key nucleotide variations of a gene lead to amino acid sequence variations, subsequently the three-dimensional protein structure variations, which results in the changes of substrate specificity. Although it’s not completely accurate to functional predict the substrate specificities of gibberellin oxidases only by amino acid sequences, it’s the major methodology for studies of plant gene families, and are widely used in most studies (He et al., 2019; Ci et al., 2021; Du et al., 2022). Therefore, the classification of the *TaGAox* genes is reliable, though their substrate specificities need further research.

The 63 *TaGAox* genes were unevenly distributed on 21 wheat chromosomes. There were two pair tandem repeat genes and 72 pairs segmental duplications, which indicated that the gene duplications played an important role in the amplification of *TaGAox* genes (Xu et al., 2020), and there were functional redundancy among these genes (Panchy, Lehti-Shiu & Shiu, 2016). Phylogenetic tree analysis divided the TaGAox proteins into seven distinct subgroups (Fig. 1), which suggested the similarities of the gene structures and functions. Most of the TaGAox proteins clustered together with or close to OsGAoxs and ZmGAoxs and far away from AtGAoxs. This means that wheat, rice and maize are close relatives. There were no Arabidopsis GAox proteins in GA7ox (V) subgroup (Fig. 1). Apparently, some *TaGAox* genes evolved independently after differentiation of monocots and dicots. Gene structure analysis discovered that most *TaGAox* genes in the same subfamily had similar exon/intron structures, most of the genes contained two or three exons, and the result was similar to GAox genes in other species, such as *Setaria italica*, *Sorghum bicolor*, *Hordeum vulgare*, *Brachypodium distachyon*, maize, and rice (Zhang et al., 2022; Ci et al., 2021). Obviously, the structures of *GAox* genes in the same subgroup are conserved, which imply their similar biological functions.

The last step of bioactive GAs synthesis is catalyzed by GA20oxs and GA3oxs to convert GA_12_ and GA_53_ into active GAs (Yamaguchi, 2008), degradation of active C_19_-GAs and C_20_-GAs by GA2oxs through 2β-hydroxylation yields inactive GA products (Schomburg et al., 2003; Otani et al., 2010). Most common motifs of TaGAox proteins were shared by the seven GAox subgroups. However, some motifs of TaGAox proteins had been lost or added in the domains of 2OG-FeII oxygenase and DIOX_N, which might lead to gene functional changes (Fig. 2). For example, motif 4 were absent in TaGA7ox-D2, motif 5 was found in GA2ox. The motif 10 can distinguish the C_19_ GA2ox and C_20_ GA2ox subgroups. It was found that the conserved sequence LPWKET of GA20ox was located in motif 8 (Table S4) (Huang et al., 2015; Pan et al., 2017). Compared with TaGA20ox (VI), TaGA7ox (V) lacks motif 8, suggesting that it may have different oxidase activities. In summary, some unique motifs existed only in specific families. To better understand the functions of TaGAoxs, the biological functions of these special motifs need to be characterized further.

### Signal transduction pathway of GAs is disturbed in *dmc*

GA signal transduction is a series of responses induced by cells after GA stimulation. Among them, GA-GID1-DELLA tricomplex plays an important role in the induction of plant growth and germination (Jiang & Fu, 2007). GA signal is perceived by GA receptor GIBBERELLIN INSENSITIVE DWARF1 (GID1), and regulate gene expression by promoting degradation of the transcriptional regulator DELLA proteins (Murase et al., 2008). In plants, when GA levels decreased, GA did not bind to GID1, so that DELLA gene inhibited the expression of GA responsive genes, thereby limited plant growth. When the GA level increased, GA and GID1 were combined to further GA-GID1-DELLA tricomplex. DELLA protein was hydrolyzed by ubiquitin-proteasome. The degradation of DELLA relieved the inhibition of GA response genes, and the plants showed normal GA response. DELLA can also induce the expression of upstream GA biosynthesis genes and GA receptors by feedback regulation (Wang & Deng, 2014). The *Rht1* gene is a gain-of-function allele caused by an N-terminal truncation near the DELLA domain and *Rht1* plants produce much more productive tillers (Kertesz, Flintham & Gale, 1991; Velu et al., 2017). Our studies had shown that DELLA and *PIF3* were involved in GA signaling pathway and were highly expressed (Fig. S3). E3 ubiquitin-protein ligase were also lowly expressed in *dmc* (He et al., 2018; Li et al., 2016). It is speculated that the decrease of sensitivity to GAs is one of the factors constraining tillering in *dmc*.

### Exogenous GA_3_ affects the expressions of *TaGAox* genes

Studies have shown that GA is one of the main hormones regulating stem elongation (Peng et al., 1999; Schomburg et al., 2003; Yamaguchi, 2008). The *semidwarf* rice *sd1* is caused by the loss of function of the *OsGA20ox2* gene. The precursor GA_53_ accumulates in the stems of *sd1*, the content of GA_1_ in *sd1* is lower than that in tall lines (Spielmeyer, Ellis & Chandler, 2002). This study found that the expression levels of *TaGA20ox-A2* and *TaGA20ox-B2* in *dmc* were lower than that in Guomai 301 (Figs. 6, 7). Therefore, we speculated that homologous genes of *TaGA20ox2* might be related to dwarfing of *dmc*. Plants can regulate GA balance by regulating expressions of *GAox* genes. In some crops, exogenous GA reduces the expressions of *GA20oxs* and *GA3oxs*, and increases the expressions of *GA2oxs* (He et al., 2019; Ci et al., 2021). The transcriptional analysis showed that the low expression levels of *TaGA20ox-A2, TaGA20ox-B2*, *TaGA2ox-D7* and *TaGA3ox-A2*, and the high expression levels of *TaGA20ox*-*A. TaGA2ox-10A* and *TaGA2ox-10B* might be the feedback regulation of GA_3_ (Fig. 6B). qRT-PCR showed that GA_3_ treatment affected the expression of *TaGAoxs*. In *dmc*, *TaGA2oxs* were up-regulated after GA treatment, and GA2ox played a role in maintaining GA balance in GA synthesis and metabolism in plants (Thomas, Phillips & Hedden, 1999). *TaGA3oxs* were basically down-regulated by exogenous GA_3_, while *TaGA20oxs* showed different trends. Among them, *TaGA7ox-D1*, *TaGA2ox10*, *TaGA2ox-A16* were most significantly regulated by GA_3_ (Fig. 7). *TaGA7ox-D1* was highly expressed after GA_3_ treatment, but no GA responsive element was detected within its promoter. It may be that there are GA responsive elements outside its 2,000 bp promoter region, or there are other regulatory modes to promote the expression of *TaGA7ox-D1*. In summary, *TaGA20ox-A2*, *TaGA20ox-B2*, *TaGA7ox1*, *TaGA2ox10*, *TaGA2ox-A16* and *TaGA3ox-A2* may play a major role in regulating the level of bioactive GAs. But how do they regulate wheat tillering needs further research.

### GA_3_ can promote wheat tillering

Studies indicate that GAs has a certain effect on plant tillering or branching. GAs negatively regulates tiller-related genes *OsH1* and *TB1* in rice, thus regulating the occurrence of tiller (Lo et al., 2008). In the tall fescue, GAs may inhibit tiller development by expressing *FaTB1* in axillary buds (Zhuang et al., 2019). It is demonstrated that gibberellin synthesis inhibitor paclobutrazol (PBZ) can promote wheat tiller formation (Assuero et al., 2012). These results suggest that high levels of active GAs in plants inhibit branching or tillering. GA_3_ can inhibit the growth of tiller buds by controlling the content of IAA or cytokinin (CTK) in plants (Liu et al., 2011). Studies have shown that GA_3_ application can inhibit the growth of wheat tiller when wheat tiller buds begin to elongate (Cai et al., 2013). However, some researches have demonstrated that application of different concentrations of GA_3_ can increase the number of wheat tillers (Islam & Mehraj, 2014). Similarly, GA_3_ can stimulate tiller development in palmarosa (*Cymbopogon martinii*) (Khan et al., 2015). In this study, continuous application of GA_3_ from the second leaf stage could promote tillering of Guomai 301, but it had no obvious effect on tillering of *dmc* (Fig. 8). It was speculated that wheat at different stages had different responses to GAs. Our previous studies showed that the contents of GAs were significantly higher, and the contents of IAA were significantly lower in the tiller buds of *dmc* (An et al., 2019). These data demonstrated that GA signaling was involved in regulating wheat tillering, but the GA signaling pathway was disturbed in *dmc*.

### *TaGA7ox-A1* can promote wheat tillering

The plant overexpression vector of pCAMBIA1304-CaMV35S:TaGA7ox-A1 was constructed and transferred into *Arabidopsis*. The primary functional analysis showed that overexpression of *TaGA7ox-A1* could significantly increase the branch numbers of the transgenic Arabidopsis plants (Fig. S4; Z. Jiao, 2020, unpublished data). Another study found that overexpression of *OsGA20ox2* promoted plant height and tiller number (Qiao et al., 2013). In switchgrass (*Panicum virgatum* L.), overexpression of *ZmGA20ox* promoted tiller number (Do et al., 2016). In this study, overexpression of *TaGA7ox-A1* significantly increased the tiller numbers of the transgenic wheat plants (Figs. 9, S2). The tiller number was positive correlated with the expression level of *TaGA7ox-A1* (*r* = 0.613, *P* < 0.05). This result demonstrated that *TaGA7ox-A1* could promote wheat tillering. However, the accurate molecular mechanism needs further study.

## Conclusions

A total of 63 *TaGAox* genes distributed on 21 wheat chromosomes were identified. *TaGAox* genes belong to seven subgroups. The promoter regions of *TaGAoxs* contained a large number of *cis*-acting elements related to plant growth, hormone signaling pathway and stress response. Segmental duplication played a major role in *TaGAoxs* amplification. The *TaGAox* genes are tissue-specifically expressed. Genes of *TaGA7ox-A1*, *TaGA20ox-A1*, *TaGA20ox-B1*, *TaGA2ox4* and *TaGA3ox-2* played basic roles during wheat tillering. The abnormal expressions of *TaGA20ox-A2*, *TaGA20ox-B2*, *TaGA3ox-A2*, *TaGA2ox10* and *TaGA20ox-A4* were involved in the synthesis and metabolism of GAs in *dmc* tiller primordia, thereby affected tiller formation. The expressions of *TaGAoxs* were significantly affected by exogenous GA_3_. Exogenous GA_3_ significantly promoted tillering of Guomai 301, but the GA pathways were disturbed in *dmc*. Overexpression *TaGA7ox-A1* promoted the transgenic wheat tillering.

## Supplemental Information

10.7717/peerj.15924/supp-1 Supplemental Information 1 Identification of *TaGA7ox-A1*-OE transgenic lines. (A) Identification analysis of TaGA7ox-A1-OE transgenic lines by genomic PCR. (B) Genomic PCR multiple sequence alignment of TaGA7ox-A1-OE transgenic lines. M: Molecular weight marker 2000; WT: wild type Guomai 301; RP: recombinant plasmid containing TaGA7ox-A1. pC1304-*TaGA7ox-A1*: sequence of the recombinant plasmid containing TaGA7ox-A1. Red boxes: TaGA7ox-A1 gene sequence; Blue lines: vector sequence; OE-L349 and OE-L353: transgenic lines.

10.7717/peerj.15924/supp-2 Supplemental Information 2 Phenotypes of the T_0_ *TaGAox-A1*-OE transgenic lines at different tillering stages. A1-C1: Before tillering; A2-C2: tillering stage; A3-C3: After tillering; 1-21: *TaGAox-A1*-OE transgenic lines. WT1-3: wild type Guomai 301.

10.7717/peerj.15924/supp-3 Supplemental Information 3 Plant hormone signal transduction (ko04075) in KEGG. Red represents the genes with high expression levels in *dmc*, green represents the genes with low expression levels in *dmc*, and blue represents the genes with both low and high expression levels in *dmc*.

10.7717/peerj.15924/supp-4 Supplemental Information 4 Phenotypes of the *TaGA7ox-A1-OE* transgenic Arabidopsis plants. WT has only one fruit branch (A: white arrows) and no branch (B: blue arrows); the transgenic lines have 3–7 fruit branches (A: red arrows) and branches (B: red arrows).

10.7717/peerj.15924/supp-5 Supplemental Information 5 The expression levels of TaGAox genes in different organs/tissues of Chinese Spring.

10.7717/peerj.15924/supp-6 Supplemental Information 6 DNA sequences of the primers used in qRT-PCR.

10.7717/peerj.15924/supp-7 Supplemental Information 7 The basic information of GAoxs in wheat.

10.7717/peerj.15924/supp-8 Supplemental Information 8 The conserved motifs in wheat GAox proteins.

10.7717/peerj.15924/supp-9 Supplemental Information 9 The duplication gene pairs of GAoxs in wheat genome.

10.7717/peerj.15924/supp-10 Supplemental Information 10 One-to-one orthologous relationships of TaGAoxs.

10.7717/peerj.15924/supp-11 Supplemental Information 11 One-to-one orthologous relationships of GAoxs between wheat and other species.

10.7717/peerj.15924/supp-12 Supplemental Information 12 The expression levels of TaGAox genes in WT (T01, T02 and T03) and dmc (T04, T05 and T06).

10.7717/peerj.15924/supp-13 Supplemental Information 13 Average of number tillers and qRT-PCR analysis of TaGA7ox-A1-OE overexpression lines.

10.7717/peerj.15924/supp-14 Supplemental Information 14 The raw data for qPT-PCR.

## Additional Information and Declarations

### Data Availability

The following information was supplied regarding data availability: The raw measurements are available in the Supplemental Files.

## References

[ref-1] Agharkar M, Lomba P, Altpeter F, Zhang HN, Kenworthy K, Lange T (2007). Stable expression of *AtGA2ox1* in a low-input turfgrass (*Paspalum notatum* Flugge) reduces bioactive gibberellin levels and improves turf quality under field conditions. Plant Biotechnology Journal.

[ref-2] An JH, Niu H, Ni YJ, Jiang YM, Zheng YX, He RS, Li JC, Jiao ZX, Zhang J, Li HJ, Li QY, Niu JS (2019). The miRNA-mRNA networks involving abnormal energy and hormone metabolisms restrict tillering in a wheat mutant *dmc*. International Journal of Molecular Sciences.

[ref-3] Ashikari M, Sasaki A, Ueguchi-Tanaka M, Itoh H, Nishimura A, Datta S, Ishiyama K, Saito T, Kobayashi M, Khush GS, Kitano H, Matsuoka M (2002). Loss-of-function of a rice gibberellin biosynthetic gene, GA20 oxidase (*GA20ox-2*), led to the rice ‘green revolution’. Breeding Science.

[ref-4] Assuero SG, Lorenzo M, Pérez Ramírez NM, Velázquez LM, Tognetti JA (2012). Tillering promotion by paclobutrazol in wheat and its relationship with plant carbohydrate status. New Zealand Journal of Agricultural Research.

[ref-5] Cai T, Meng XP, Liu XL, Liu TN, Wang H, Jia ZK, Yang DQ, Ren XL (2018). Exogenous hormonal application regulates the occurrence of wheat tillers by changing endogenous hormones. Frontiers in Plant Science.

[ref-6] Cai T, Xu HC, Yin YP, Yang WB, Peng DL, Ni YL, Xu CL, Yang DQ, Wang ZL (2013). Mechanisms of tiller occurrence affected by exogenous IAA, GA_3_, and ABA in wheat with different spike-types. Acta Agronomica Sinica.

[ref-7] Cao Y, Hu G, Zhuang MJ, Yin J, Wang X (2021). Molecular cloning and functional characterization of *TaIRI9* gene in wheat (*Triticum aestivum* L.). Gene.

[ref-8] Chen CJ, Chen H, Zhang Y, Thomas HR, Frank MH, He Y, Xia R (2020). TBtools: an integrative toolkit developed for interactive analyses of big biological data. Molecular Plant.

[ref-9] Cheng J, Ma JJ, Zheng XB, Lv HL, Zhang MM, Tan B, Ye X, Wang W, Zhang LL, Li ZQ, Feng JC (2021). Functional analysis of the gibberellin 2-oxidase gene family in peach. Frontiers in Plant Science.

[ref-10] Ci JB, Wang XY, Wang Q, Zhao FX, Yang W, Cui XY, Jiang LY, Ren XJ, Yang WG (2021). Genome-wide analysis of gibberellin-dioxygenases gene family and their responses to GA applications in maize. PLOS ONE.

[ref-11] Do PT, De Tar JR, Lee H, Folta MK, Zhang ZJ (2016). Expression of *ZmGA20ox* cDNA alters plant morphology and increases biomass production of switchgrass (*Panicum virgatum* L.). Plant Biotechnology Journal.

[ref-12] Dong FS, Liu YW, Lu MY, Zhou S, Yang F (2018). Genetic characters of progenies of transgenic wheat by shoot apical point transformation. Acta Agriculturae Borealioccidentalis Sinica.

[ref-13] Du LL, Li SM, Ding L, Cheng XX, Kang ZS, Mao HD (2022). Genome-wide analysis of trehalose-6-phosphate phosphatases (TPP) gene family in wheat indicates their roles in plant development and stress response. BMC Plant Biology.

[ref-14] Elfving DC, Visser DB, Henry JL (2011). Gibberellins stimulate lateral branch development in young sweet cherry trees in the orchard. International Journal of Fruit Science.

[ref-15] Frisse A, Pimenta MJ, Lange T (2003). Expression studies of gibberellin oxidases in developing pumpkin seeds. Plant Physiology.

[ref-16] Han FM, Zhu BG (2011). Evolutionary analysis of three gibberellin oxidase genes in rice, Arabidopsis, and soybean. Gene.

[ref-17] He HH, Liang GP, Lu SX, Wang PP, Liu T, Ma ZH, Zuo CW, Sun XM, Chen BH, Mao J (2019). Genome-wide identification and expression analysis of GA2ox, GA3ox, and GA20ox are related to gibberellin oxidase genes in grape (*Vitis Vinifera* L.). Genes.

[ref-18] He YR, Liu W, Huang ZH, Huang JS, Xu YH, Zhang QN, Hu J (2022). Genome-wide analysis of the rice gibberellin dioxygenases family genes. Agronomy.

[ref-19] He RS, Ni YJ, Li JC, Jiao ZX, Zhu XX, Jiang YM, Li QY, Niu JS (2018). Quantitative changes in the transcription of phytohormone-related genes: some transcription factors are major causes of the wheat mutant *dmc* not tillering. International Journal of Molecular Sciences.

[ref-20] Hedden P (2020). The current status of research on gibberellin biosynthesis. Plant and Cell Physiology.

[ref-21] Hedden P, Phillips AL (2000). Gibberellin metabolism: new insights revealed by the genes. Trends in Plant Science.

[ref-22] Hedden P, Thomas SG (2012). Gibberellin biosynthesis and its regulation. Biochemical Journal.

[ref-23] Hernández-García J, Briones-Moreno A, Blázquez MA (2021). Origin and evolution of gibberellin signaling and metabolism in plants. Seminars in Cell & Developmental Biology.

[ref-24] Holub EB (2001). The arms race is ancient history in Arabidopsis, the wildflower. Nature Reviews Genetics.

[ref-25] Honi U, Amin MR, Kabir SMT, Bashar KK, Moniruzzaman M, Jahan R, Jahan S, Haque MS, Islam S (2020). Genome-wide identification, characterization and expression profiling of gibberellin metabolism genes in jute. BMC Plant Biology.

[ref-26] Hu JH, Mitchum MG, Barnaby N, Ayele BT, Ogawa M, Nam E, Lai WC, Hanada A, Alonso JM, Ecker JR, Swain SM, Yamaguchi S, Kamiya Y, Sun TP (2008). Potential sites of bioactive gibberellin production during reproductive growth in Arabidopsis. The Plant Cell.

[ref-27] Hu LF, Wang PK, Hao ZD, Lu Y, Xue GX, Cao ZJ, Qu HX, Cheng TL, Shi JS, Chen JH (2021). Gibberellin oxidase gene family in L. *chinense*: genome-wide identification and gene expression analysis. International Journal of Molecular Sciences.

[ref-28] Huang Y, Wang X, Ge S, Rao GY (2015). Divergence and adaptive evolution of the gibberellin oxidase genes in plants. BMC Evolutionary Biology.

[ref-29] Hurst LD (2002). The Ka/Ks ratio: diagnosing the form of sequence evolution. Trends in Genetics.

[ref-30] Islam S, Mehraj H (2014). Growth and yield of wheat as influenced by GA_3_ concentrations. International Journal of Business Social and Scientific Research.

[ref-31] Jiang CF, Fu XD (2007). GA action: turning on de-DELLA repressing signaling. Current Opinion in Plant Biology.

[ref-32] Kawai Y, Ono E, Mizutani M (2014). Evolution and diversity of the 2-oxoglutarate-dependent dioxygenase superfamily in plants. The Plant Journal: For Cell and Molecular Biology.

[ref-33] Kertesz Z, Flintham JE, Gale MD (1991). Effects of *Rht* dwarfing genes on wheat grain yield and its components under Eastern European conditions. Cereal Research Communications.

[ref-34] Khan AF, Mujeeb F, Aha F, Farooqui A (2015). Effect of plant growth regulators on growth and essential oil content in palmarosa (*Cymbopogon martinii*). Asian Journal of Pharmaceutical and Clinical Research.

[ref-35] Kumagai Y, Liu YL, Hamada H, Luo WF, Zhu JH, Kuroki M, Nagira Y, Taoka N, Katoh E, Imai R (2022). Introduction of a second “Green Revolution” mutation into wheat via in planta CRISPR/Cas9 delivery. Plant Physiology.

[ref-36] Lange T, Krämer C, Lange MJP (2020). The class III gibberellin 2-oxidases *AtGA2ox9* and *AtGA2ox10* contribute to cold stress tolerance and fertility. Plant Physiology.

[ref-37] Lange T, Lange MJP (2020). The multifunctional dioxygenases of gibberellin synthesis. Plant & Cell Physiology.

[ref-38] Lange MJP, Liebrandt A, Arnold L, Chmielewska SM, Felsberger A, Freier E, Heuer M, Zur D, Lange T (2013). Functional characterization of gibberellin oxidases from cucumber, *Cucumis sativus* L. Phytochemistry.

[ref-39] Lange MJP, Szperlinski M, Kalix L, Lange T (2020). Cucumber gibberellin 1-oxidase/desaturase initiates novel gibberellin catabolic pathways. Journal of Biological Chemistry.

[ref-40] Li JC, Jiang YM, Zhang J, Ni YJ, Jiao ZX, Li HJ, Wang T, Zhang PP, Guo WL, Li L, Liu HJ, Zhang HR, Li QY, Niu JS (2021). Key *auxin response factor* (*ARF*) genes constraining wheat tillering of mutant *dmc*. PeerJ.

[ref-41] Li KL, Yu RB, Fan LM, Wei N, Chen HD, Deng XW (2016). DELLA-mediated PIF degradation contributes to coordination of light and gibberellin signalling in Arabidopsis. Nature Communications.

[ref-42] Lin QB, Wu FQ, Sheng PK, Zhang Z, Zhang X, Guo XP, Wang JL, Cheng ZJ, Wang J, Wang HY, Wan JM (2015). The SnRK_2_-APC/C^(TE)^ regulatory module mediates the antagonistic action of gibberellic acid and abscisic acid pathways. Nature Communications.

[ref-43] Lin QB, Zhang Z, Wu FQ, Feng M, Sun Y, Chen WW, Cheng ZJ, Zhang X, Ren YL, Lei CL, Zhu SS, Wang J, Zhao ZC, Guo XP, Wang HY, Wan JM (2020). The APC/C^TE^ E3 ubiquitin ligase complex mediates the antagonistic regulation of root growth and tillering by ABA and GA. The Plant Cell.

[ref-44] Liu X, Hou XL (2018). Antagonistic regulation of ABA and GA in metabolism and signaling pathways. Frontiers in Plant Science.

[ref-45] Liu Y, Wang QS, Ding YF, Li GH, Xu JX, Wang SH (2011). Effects of external ABA, GA_3_ and NAA on the tiller bud outgrowth of rice is related to changes in endogenous hormones. Plant Growth Regulation.

[ref-46] Livak KJ, Schmittgen TD (2001). Analysis of relative gene expression data using real-time quantitative PCR and the 2^−ΔΔCT^ method. Methods.

[ref-47] Lo SF, Yang SY, Chen KT, Hsing YL, Zeevaart JA, Chen LJ, Yu SM (2008). A novel class of gibberellin 2-oxidases control semidwarfism, tillering, and root development in rice. The Plant Cell.

[ref-48] Macmillan J, Takahashi N (1968). Proposed procedure for the allocation of trivial names to the gibberellins. Nature.

[ref-49] Martínez-Bello L, Moritz T, López-Díaz I (2015). Silencing C_19_-GA2-oxidases induces parthenocarpic development and inhibits lateral branching in tomato plants. Journal of Experimental Botany.

[ref-50] Murase K, Hirano Y, Sun TP, Hakoshima T (2008). Gibberellin-induced DELLA recognition by the gibberellin receptor GID1. Nature.

[ref-51] Nakamura H, Xue YL, Miyakawa T, Hou F, Qin HM, Fukui K, Shi X, Ito E, Ito S, Park SH, Miyauchi Y, Asano A, Naoya T, Ueda T, Tanokura M, Asami T (2013). Molecular mechanism of strigolactone perception by *DWARF14*. Nature Communications.

[ref-52] Ni J, Gao CC, Chen MS, Pan BZ, Ye KQ, Xu ZF (2015a). Gibberellin promotes shoot branching in the perennial woody plant *Jatropha curcas*. Plant and Cell Physiology.

[ref-53] Ni YJ, Zhu PP, Liu HJ, Hu X, Li QY, Niu JS (2015b). Construction and analysis of EMS induced mutant library of new wheat cultivar Guomai 301. Journal of Henan Agricultural Sciences.

[ref-54] Otani M, Yoon JM, Park SH, Asami T, Nakajima M (2010). Screening and characterization of an inhibitory chemical specific to Arabidopsis gibberellin 2-oxidases. Bioorganic & Medicinal Chemistry Letters.

[ref-55] Pan C, Tian KH, Ban QY, Wang LG, Sun QL, He Y, Yang YF, Pan YT, Li YY, Jiang JY (2017). Genome-wide analysis of the biosynthesis and deactivation of gibberellin-dioxygenases gene family in *Camellia sinensis* (L.) O. Kuntze. Genes.

[ref-56] Panchy N, Lehti-Shiu M, Shiu SH (2016). Evolution of gene duplication in plants. Plant Physiology.

[ref-57] Pearce S, Huttly AK, Prosser IM, Li YD, Vaughan SP, Gallova B, Patil A, Coghill JA, Dubcovsky J, Hedden P, Phillips AL (2015). Heterologous expression and transcript analysis of gibberellin biosynthetic genes of grasses reveals novel functionality in the GA3ox family. BMC Plant Biology.

[ref-58] Peng JR, Richards DE, Moritz T, Cano-Delgado A, Harberd NP (1999). Extragenic suppressors of the Arabidopsis gai mutation alter the dose-response relationship of diverse gibberellin responses. Plant Physiology.

[ref-59] Qiao F, Wang CL, Chen Z, Geng GG (2013). Influence on plant architecture through the over expression of OsGA20ox2 in rice. Journal of China Agricultural University.

[ref-60] Rinne PLH, Paul LK, Vahala J, Kangasjarvi J, van der Schoot C (2016). Axillary buds are dwarfed shoots that tightly regulate GA pathway and GA-inducible 1,3-beta-glucanase genes during branching in hybrid aspen. Journal of Experimental Botany.

[ref-61] Schomburg FM, Bizzell CM, Lee DJ, Zeevaart JA, Amasino RM (2003). Overexpression of a novel class of gibberellin 2-oxidases decreases gibberellin levels and creates dwarf plants. The Plant Cell.

[ref-62] Spielmeyer W, Ellis MH, Chandler PM (2002). *Semidwarf* (*sd-1*), “green revolution” rice, contains a defective gibberellin 20-oxidase gene. Proceedings of the National Academy of Sciences of the United States of America.

[ref-63] Spielmeyer W, Ellis M, Robertson M, Ali S, Lenton JR, Chandler PM (2004). Isolation of gibberellin metabolic pathway genes from barley and comparative mapping in barley, wheat and rice. Theoretical and Applied Genetics.

[ref-64] Sun H, Pang B, Yan J, Wang T, Wang L, Chen C, Li Q, Ren Z (2018). Comprehensive analysis of cucumber gibberellin oxidase family genes and functional characterization of *CsGA20ox1* in root development in *Arabidopsis*. International Journal of Molecular Sciences.

[ref-65] Thomas SG, Phillips AL, Hedden P (1999). Molecular cloning and functional expression of gibberellin 2-oxidases, multifunctional enzymes involved in gibberellin deactivation. Proceedings of the National Academy of Sciences of the United States of America.

[ref-66] Tian XL, Xia XC, Xu DG, Liu YQ, Xie L, Hassan MA, Song J, Li FJ, Wang DS, Zhang Y, Hao YF, Li GY, Chu CC, He ZH, Cao SH (2022). *Rht24b*, an ancient variation of *TaGA2ox-A9*, reduces plant height without yield penalty in wheat. New Phytologist.

[ref-67] Urbanova T, Leubner-Metzger G (2016). Gibberellins and seed germination. Annual Plant Reviews.

[ref-68] Velu G, Singh RP, Huerta J, Guzmán C (2017). Genetic impact of *Rht* dwarfing genes on grain micronutrients concentration in wheat. Field Crops Research.

[ref-69] Wang YJ, Deng DX (2014). Molecular basis and evolutionary pattern of GA-GID1-DELLA regulatory module. Molecular Genetics and Genomics.

[ref-70] Wang ZQ, Shi HR, Yu SF, Zhou WL, Li J, Liu SH, Deng M, Ma J, Wei YM, Zheng YL, Liu YX (2019). Comprehensive transcriptomics, proteomics, and metabolomics analyses of the mechanisms regulating tiller production in low-tillering wheat. Theoretical and Applied Genetics.

[ref-71] Wang YP, Tang HB, DeBarry JD, Tan X, Li JP, Wang XY, Lee TH, Jin HZ, Marler B, Guo H, Kissinger JC, Paterson AH (2012). *MCScanX*: a toolkit for detection and evolutionary analysis of gene synteny and collinearity. Nucleic Acids Research.

[ref-72] Xu Q, Krishnan S, Merewitz E, Xu JH, Huang BR (2016). Gibberellin-regulation and genetic variations in leaf elongation for tall fescue in association with differential gene expression controlling cell expansion. Scientific Reports.

[ref-73] Xu ZC, Pu XD, Gao RR, Demurtas OC, Fleck SJ, Richter M, He CN, Ji AJ, Sun W, Kong JQ, Hu KZ, Ren FM, Song JJ, Wang Z, Gao T, Xiong C, Yu HY, Xin TY, Aibert VA, Giuliano G, Chen SL, Song JY (2020). Tandem gene duplications drive divergent evolution of caffeine and crocin biosynthetic pathways in plants. BMC Biology.

[ref-74] Yamaguchi S (2006). Gibberellin biosynthesis in Arabidopsis. Phytochemistry Reviews.

[ref-75] Yamaguchi S (2008). Gibberellin metabolism and its regulation. Annual Review of Plant Biology.

[ref-76] Yamaguchi S, Kamiya Y (2001). Gibberellins and light-stimulated seed germination. Journal of Plant Growth Regulation.

[ref-77] Yang L, Liu Y, Xiang Y, Sun XJ, Yan JW, Zhang AY (2021). Establishment and optimization of a shoot tip-based genetic transformation system for foxtail millet. Chinese Bulletin of Botany.

[ref-78] Yang DQ, Luo YL, Kong X, Huang C, Wang ZL (2020). Interactions between exogenous cytokinin and nitrogen application regulate tiller bud growth via sucrose and nitrogen allocation in winter wheat. Journal of Plant Growth Regulation.

[ref-79] Yu H, Ito T, Zhao YX, Peng J, Kumar PR, Meyerowitz EM (2004). Floral homeotic genes are targets of gibberellin signaling in flower development. Proceedings of the National Academy of Sciences of the United States of America.

[ref-80] Zhang J, Li JC, Ni YJ, Jiang YM, Jiao ZX, Li HJ, Wang T, Zhang PP, Han MY, Li L, Liu HJ, Li QY, Niu JS (2021). Key wheat *GRF* genes constraining wheat tillering of mutant *dmc*. PeerJ.

[ref-81] Zhang CH, Nie X, Kong WL, Deng XX, Sun T, Liu XH, Li YS (2022). Genome-wide identification and evolution analysis of the gibberellin oxidase gene family in six gramineae crops. Genes.

[ref-82] Zhou W, Malabanan PB, Abrigo E (2015). *OsHox4* regulates GA signaling by interacting with DELLA-like genes and GA oxidase genes in rice. Euphytica.

[ref-83] Zhuang LL, Ge Y, Wang J, Yu JJ, Yang ZM, Huang BR (2019). Gibberellic acid inhibition of tillering in tall fescue involving crosstalks with cytokinins and transcriptional regulation of genes controlling axillary bud outgrowth. Plant Science.

