# Supplementary figures and images for: Wheat gibberellin oxidase genes and their functions in regulating tillering
Source: PeerJ. 2023 Sep 1;11:e15924. doi: 10.7717/peerj.15924 (PMC10476609; doi:10.7717/peerj.15924)

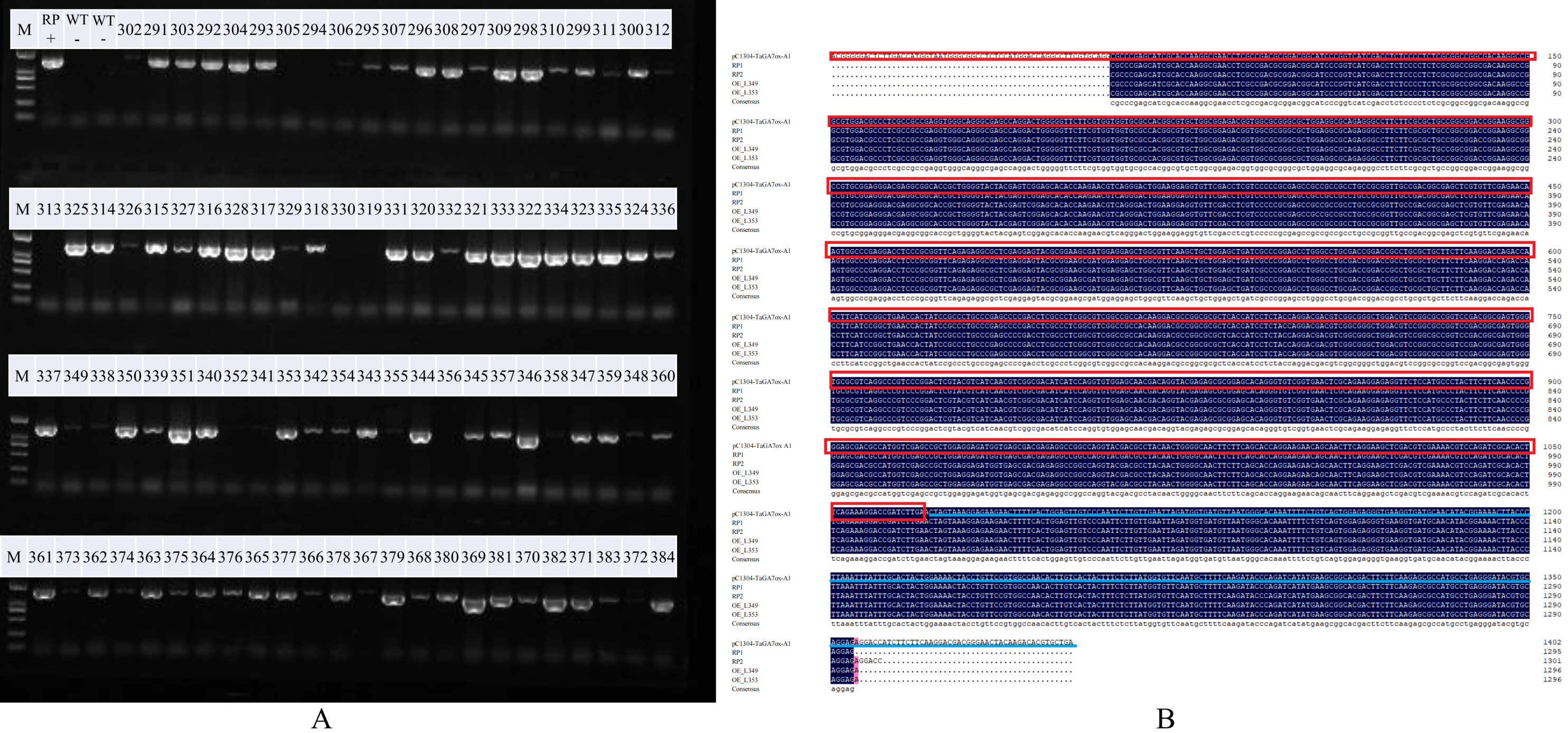

Supplement: Supplemental Information 1 — (A) Identification analysis of TaGA7ox-A1-OE transgenic lines by genomic PCR. (B) Genomic PCR multiple sequence alignment of TaGA7ox-A1-OE transgenic lines. M: Molecular weight marker 2000; WT: wild type Guomai 301; RP: recombinant plasmid containing TaGA7ox-A1. pC1304-TaGA7ox-A1: sequence of the recombinant plasmid containing TaGA7ox-A1. Red boxes: TaGA7ox-A1 gene sequence; Blue lines: vector sequence; OE-L349 and OE-L353: transgenic lines. [file peerj-11-15924-s001.png]

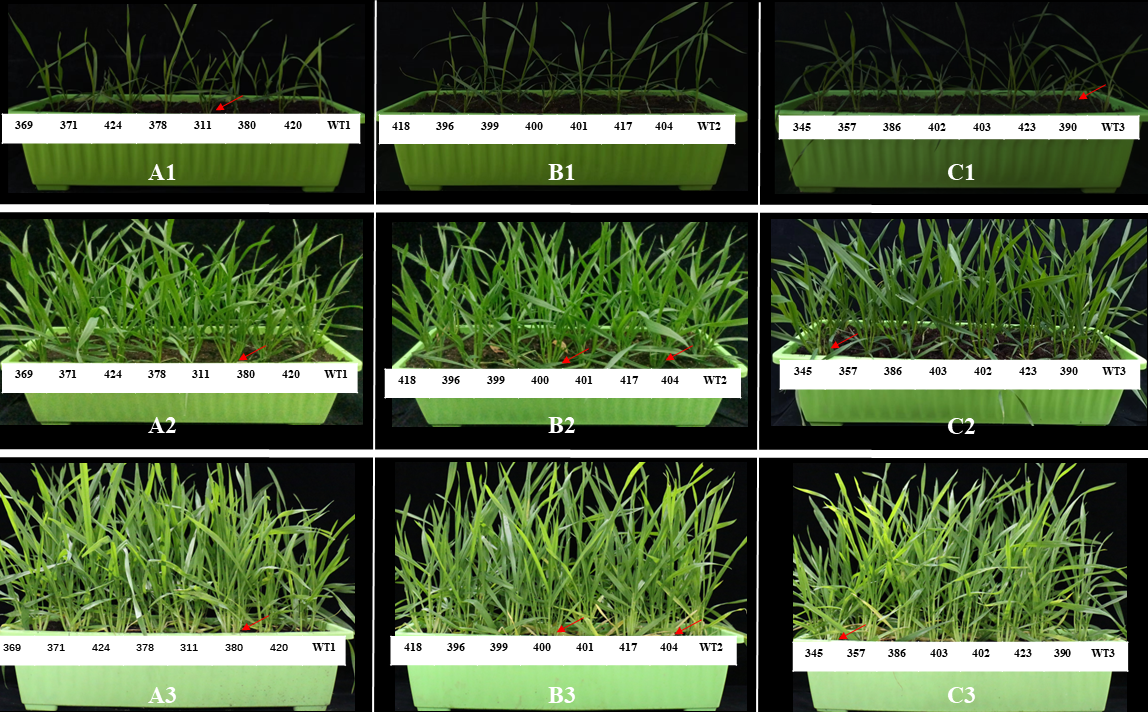

Supplement: Supplemental Information 2 — A1-C1: Before tillering; A2-C2: tillering stage; A3-C3: After tillering; 1-21: TaGAox-A1-OE transgenic lines. WT1-3: wild type Guomai 301. [file peerj-11-15924-s002.png]

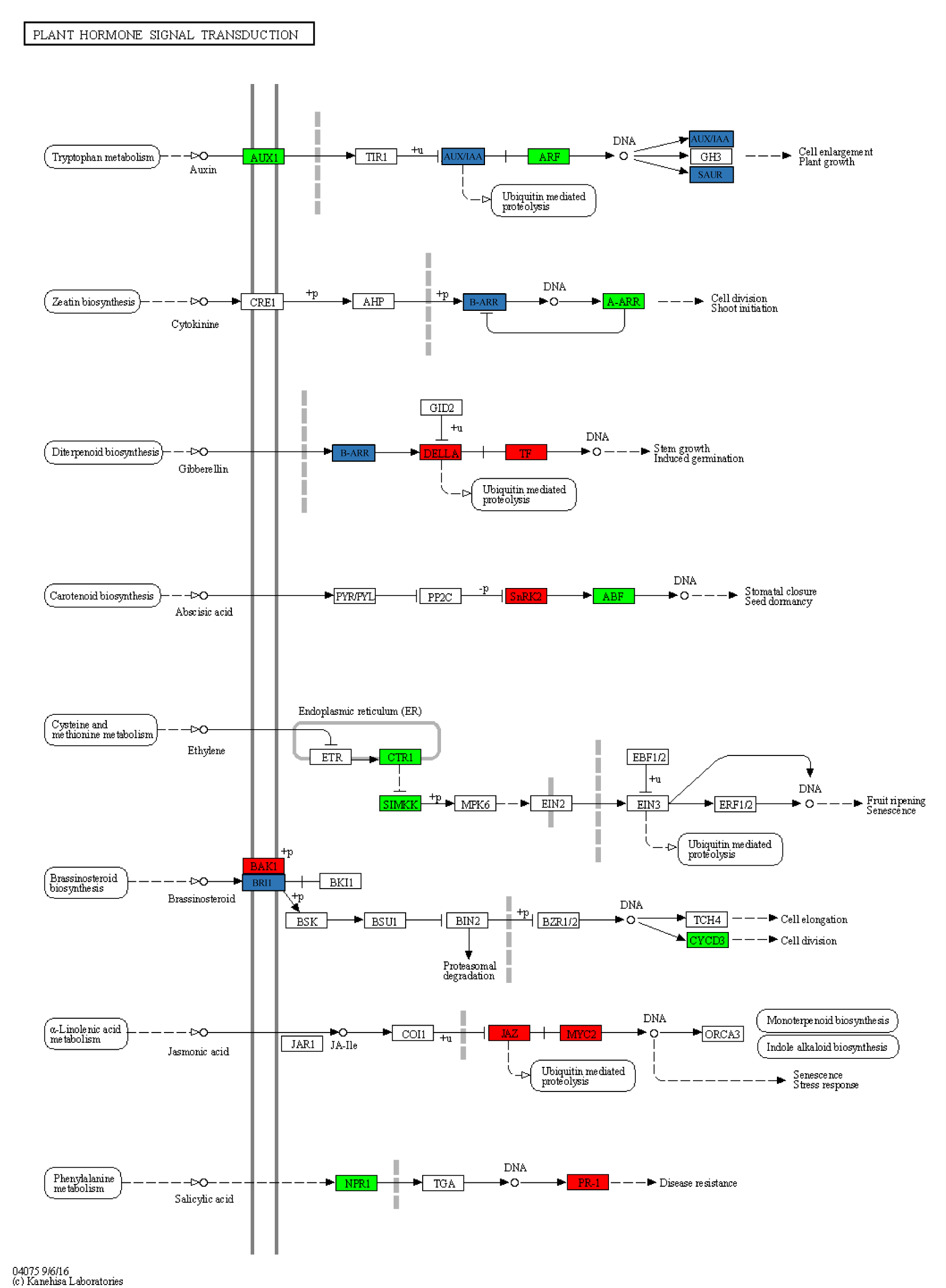

Supplement: Supplemental Information 3 — Red represents the genes with high expression levels in dmc, green represents the genes with low expression levels in dmc, and blue represents the genes with both low and high expression levels in dmc. [file peerj-11-15924-s003.png]

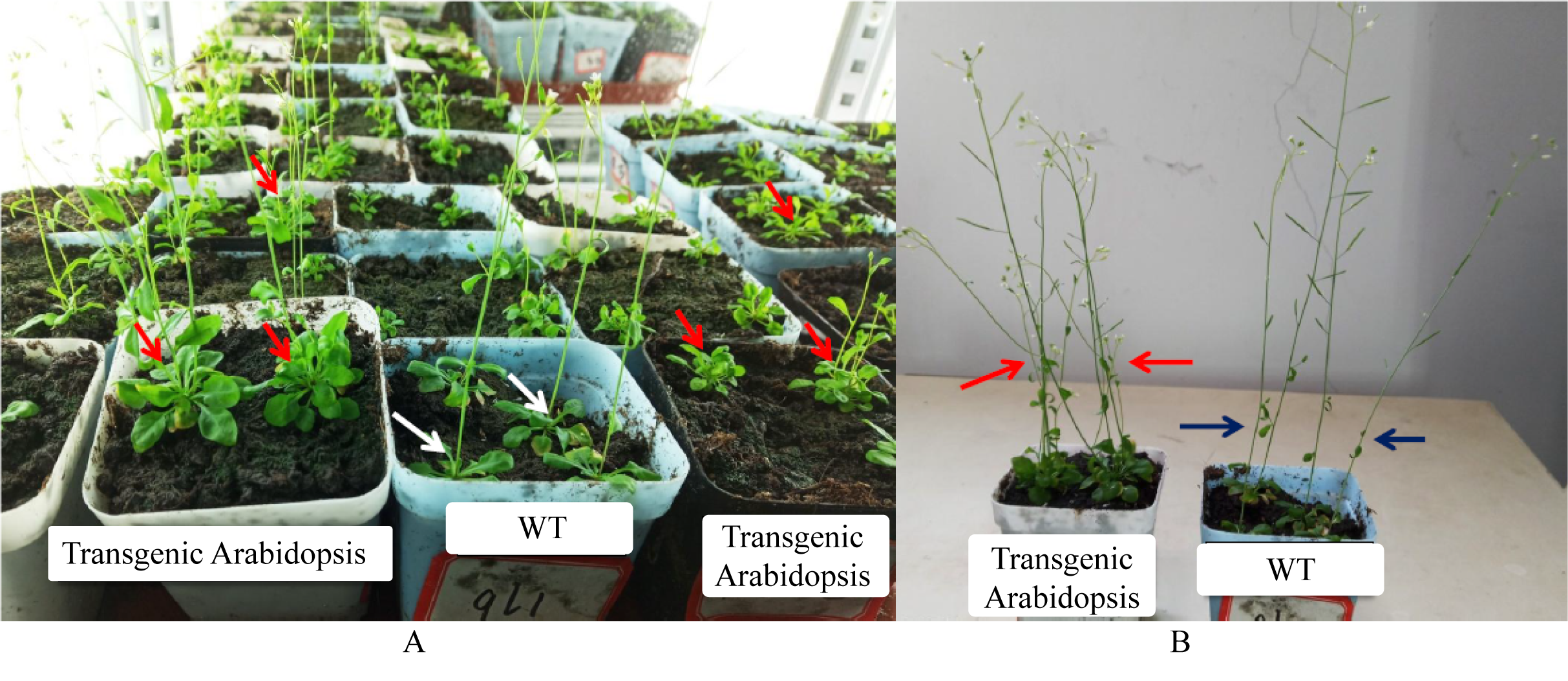

Supplement: Supplemental Information 4 — WT has only one fruit branch (A: white arrows) and no branch (B: blue arrows); the transgenic lines have 3–7 fruit branches (A: red arrows) and branches (B: red arrows). [file peerj-11-15924-s004.png]
